# Mechanisms and Production of Hypoglycaemic Peptides: Exploring the Potential of *Chlamydomonas reinhardtii*

**DOI:** 10.1002/fsn3.71790

**Published:** 2026-04-14

**Authors:** Qian Li, Keying Su, Chunmin Yang, Xiaoyan Sun, Jing Huang, Saiyi Zhong, Lai‐Hoong Cheng

**Affiliations:** ^1^ School of Food & Pharmaceutical Science and Technology Guangzhou College of Technology and Business Guangzhou China; ^2^ Food Technology Division, School of Industrial Technology Universiti Sains Malaysia Penang Malaysia; ^3^ College of Food Science and Technology Guangdong Ocean University Zhanjiang China

**Keywords:** bioactive peptides, *Chlamydomonas reinhardtii*, diabetes management, functional foods, hypoglycaemic activity

## Abstract

Diabetes, particularly type 2 diabetes mellitus (T2DM), has emerged as a major global health challenge, while currently available therapeutic drugs are frequently associated with drug resistance and adverse side effects. In recent years, bioactive peptides have attracted increasing attention as promising hypoglycaemic candidates due to their favorable safety profiles, good tolerability, and multi‐target physiological regulatory activities. As a GRAS‐designated unicellular green alga, *Chlamydomonas reinhardtii* is characterized by a high protein content, with approximately 46.9% of its dry biomass composed of protein, making it a rich source for the development of hypoglycaemic short peptides. Previous studies have shown that proteolytic products derived from *C. reinhardtii* markedly inhibit key glucose‐regulating enzymes, including α‐amylase and α‐glucosidase, exhibiting superior inhibitory potential compared with various terrestrial protein sources. Nevertheless, challenges remain in the large‐scale production of *C. reinhardtii* ‐derived hypoglycaemic peptides, precise control of the proteolytic process, and validation of their in vivo efficacy. This review summarizes the major sources of hypoglycaemic peptides, current extraction and characterization strategies, and their underlying mechanisms of action, while highlighting the application potential and key limitations of *C. reinhardtii* ‐derived hypoglycaemic peptides. Addressing these challenges is expected to facilitate the development and application of *C. reinhardtii* ‐based functional foods and nutraceuticals for diabetes management.

## Graphical abstract

This review summarizes the sources, preparation strategies, and mechanisms of hypoglycaemic peptides, with particular emphasis on the glucose‐lowering potential of *Chlamydomonas reinhardtii* . It also highlights their prospective applications and current challenges in the development of functional foods and nutraceuticals for diabetes management.

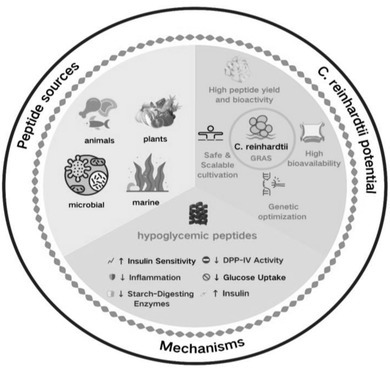

## 1 Introduction

Diabetes is a chronic metabolic disorder characterized by persistently elevated blood glucose levels, which can lead to serious health complications, including heart disease, kidney disease, blindness, and lower limb amputations (Ceriello et al. 2021). Diabetes mellitus is primarily classified into two main types: Type 1 and Type 2 diabetes. Type 2 diabetes mellitus (T2DM) accounts for over 90% of diabetes cases (Aktas 2025). Type 1 diabetes is caused by a combination of autoimmune, genetic, and environmental factors, and is characterized by the autoimmune destruction of pancreatic β‐cells, resulting in a complete lack of insulin production (Forouhi and Wareham 2018). Type 2 diabetes, more common in adults, is associated with obesity, lack of exercise, and genetic predisposition. It is characterized by insulin resistance, where the body does not use insulin efficiently or does not produce sufficient insulin (Van Heck et al. 2024).

Current diabetes treatments include insulin injections, Glucagon‐like peptide‐1 (GLP‐1) receptor agonists, and various oral medications. Oral antidiabetic drugs include alpha‐glucosidase inhibitors (AGIs) such as acarbose, miglitol, and voglibose, which lower blood glucose by inhibiting carbohydrate digestion and limiting sugar absorption in the intestines. Other drugs include biguanides (e.g., metformin), which increase insulin sensitivity by reducing hepatic glucose production, sulfonylureas (e.g., glibenclamide) that stimulate insulin secretion, thiazolidinediones (e.g., rosiglitazone) which enhance insulin sensitivity, dipeptidyl peptidase IV (DPP‐IV) inhibitors (e.g., sitagliptin) that prolong the action of endogenous GLP‐1 and Glucose‐dependent insulinotropic peptide (GIP), and sodium‐glucose co‐transporter 2 (SGLT2) inhibitors (e.g., dagliflozin) that decrease renal glucose reabsorption (Bonora et al. 2020; Khunti et al. 2024; Lebovitz 2019; Gajjar et al. 2025).

However, traditional drug therapies often face challenges such as resistance and adverse side effects. For instance, long‐term use of insulin may decrease the sensitivity of insulin receptors, leading to insulin resistance (Accili et al. 2025). Frequent use of hypoglycaemic drugs can result in side effects like bone loss, headaches, urination issues, haemorrhagic or necrotizing acute pancreatitis, upper respiratory tract infections, and gastrointestinal disturbances (Scheen 2022). Consequently, there is an increasing demand for safer and more effective oral hypoglycaemic agents.

Peptides have gained increasing attention in drug development. Bioactive peptides are short protein fragments (2–30 amino acids) generated by enzymatic hydrolysis, and their physiological activities are closely associated with their amino acid composition and sequence (Khakhariya et al. 2023). Food‐derived bioactive peptides classified based on their source (plant, animal, marine, microbial, or biosynthetic) generally offer better safety and tolerability with relatively few side effects, making them promising candidates for long‐term therapy (De Castro and Sato 2015). Several food‐derived bioactive peptides have shown hypoglycaemic effects, including those from bitter melon (Yuan et al. 2008), camel milk, buffalo milk (Khakhariya et al. 2023), rubing cheese (Li et al. 2022), beans (Valencia‐Mejía et al. 2019), cacao (Sarmadi et al. 2012), and algae (Ramos‐Romero et al. 2021; Zhao et al. 2018).

*Chlamydomonas reinhardtii* (Dangeard) (hereinafter referred to as *C. reinhardtii* ) is a unicellular green alga known for its ease of cultivation, rapid growth, and unique genetic makeup, making it suitable for various biotechnological applications. It is rich in proteins that can be enzymatically hydrolysed to produce bioactive peptides with potential health benefits. Currently, *C. reinhardtii* peptides have been reported to exhibit antioxidant, antibacterial, antiviral, antihypertensive, immunomodulatory, and anticancer activities (Bhandari et al. 2020; Ovchinnikova 2019). Given the promising reports on the extraction of hypoglycaemic peptides from algae (Leong and Chang 2024), *C. reinhardtii* is a potential source for producing hypoglycaemic peptides due to its high protein content.

This review summarizes current strategies for the production and characterization of hypoglycaemic peptides, elucidates their mechanisms in blood glucose regulation, and evaluates the therapeutic potential of *C. reinhardtii* ‐derived hypoglycaemic peptides for the treatment of diabetes mellitus.

## 2 Hypoglycaemic Bioactive Peptides and Their Mechanisms of Action

Bioactive peptides are specific protein fragments that are inactive within their parent proteins but become biologically active following enzymatic digestion, fermentation, or chemical synthesis (Tagliamonte et al. 2024; Qu et al. 2025). Once activated, these peptides interact with receptors or enzymes in the body, exhibiting various beneficial biological activities, including antimicrobial, antioxidant, antihypertensive, immunomodulatory, anticancer, and hypoglycemic effects among others (Gedif and Tkaczewska 2024).

Hypoglycemic peptides, a subset of bioactive peptides, are typically derived from food proteins. Their mechanism of action involves mimicking insulin or enhancing the body's insulin response, making them promising candidates for diabetes management (Campbell and Newgard 2021). These peptides are sourced from animals (e.g., milk, fish), plants (e.g., legumes, grains), algae, and microorganisms (e.g., yeast, *lactobacilli*). Table 1 provides a summary of the hypoglycemic effects and mechanisms of action of various food‐derived peptides across different groups.

**TABLE 1:** Recent studies on hypoglycemic peptides of natural origin (“—” indicates that no enzyme was used or that the peptide sequence was not identified).

Source of protein	Peptide preparation method	Types of enzymes used	Significant peptide sequence	Hypoglycemic mechanisms	References
Animal proteins					
Whey	Enzymatic hydrolysis	Papain	YPVEPF	Inhibits α‐amylase (IC_50_ = 3.52 mg/mL); promotes glucose uptake in HepG2 cells (0.25–0.5 mg/mL).	Li et al. (2022)
Fish ( *Trachinotus ovatus* )	Enzymatic hydrolysis	Trypsin	—	Improves random blood glucose (RBG) levels in diabetic mice; enhances insulin secretion; alleviates liver and kidney injury in STZ‐induced diabetic mice	Wan et al. (2023)
β‐Lactoglobulin	Enzymatic hydrolysis	Trypsin	VAGTWY	DPP‐IV inhibition (IC_50_ = 174 μM).	Uchida et al. (2011)
Sheep milk	Fermentation	—	—	Inhibits α‐amylase and α‐glucosidase; suppresses pro‐inflammatory cytokines in RAW 264.7 macrophages.	Pipaliya et al. (2024)
Fish ( *Siniperca chuatsi* )	Fermentation	—	EPAEAVGDWR, IPHESVDVIK, PDLSKHNNHM, PFGNTHNNFK	DPP‐IV inhibition; IC_50_ = 0.10, 2.69, 3.88, and 8.51 mM for respective peptides.	Yang et al. (2022)
Rabbit meat	Enzymatic hydrolysis	Pepsin, Flavourzyme, Alcalase, Bromelain, compound protease	Leucyl‐Leucine	DPP‐IV inhibition (IC_50_ = 99.85 ± 5.45 μM).	Hu et al. (2024)
Plant protein					
Bitter melon ( *Momordica charantia* )	Aqueous extraction	—	GHPYYSIKKS	Lowers blood glucose in alloxan‐induced diabetic mice.	Yuan et al. (2008)
Hemp (*Cannabis sativa L*)	Enzymatic hydrolysis	Alkaline protease	TGLGR, SPVI, FY, FR	Inhibits α‐amylase; regulates glucose and lipid metabolism; improves insulin resistance.	Cai et al. (2023)
Cocoa (*Theobroma cacao L*.)	Autolysis	Aspartic endoprotease; Carboxypeptidase	—	Inhibits α‐amylase; stimulates insulin secretion in BRIN‐BD11 pancreatic cells (except U5, 0.3 mg/mL).	Van Der Bruggen (2018)
Walnut ( *Juglans mandshurica* )	Enzymatic hydrolysis	Alcalase	LPLLR	Inhibits α‐amylase and α‐glucosidase; activates IRS‐1/PI3K/Akt and AMPK pathways in insulin‐resistant HepG2 cells.	Wang et al. (2020)
Soybean	Extrusion and in vitro digestion	—	LLRPPK	Inhibits α‐glucosidase; enhances SOD and GSH‐Px activity; reduces oxidative stress markers; protects liver and pancreas.	Li, Fu, et al. (2023)
Ginkgo biloba seed	Enzymatic hydrolysis	Alcalase; Bromelain; Flavourzyme	LSMSFPP, VPKIPPP, MPGPPSD	Inhibits α‐glucosidase; IC_50_ values: 454.33 ± 32.45 μM (LSMSFPP), 1446.81 ± 66.98 μM (VPKIPPP), 943.82 ± 73.10 μM (MPGPPSD).	Wang, Deng, et al. (2023)
Soybean	Enzymatic hydrolysis	Alcalase	—	Inhibits α‐amylase and α‐glucosidase; regulates gluconeogenesis‐related genes: ↓NR4A1, BTG2, FBP1, SOCS3, PTGS2, SGK1; ↑IRS1.	Xue et al. (2024)
Algae proteins					
*Spirulina platensis*	Ultrasound coupled with subcritical water extraction	—	GVPMPNK (GK), RNPFVFAPTLLTVAAR (RR), LRSELAAWSR (LR)	Inhibits α‐amylase and α‐glucosidase; IC_50_ (α‐amylase): 236.2, 1077.6, 313.6 μg/mL (α‐glucosidase): 151.5, 164.5, 134.2 μg/mL; DPP‐IV: 192.3, 181.2, 167.3 μg/mL (GK, RR, LR respectively)	Hu et al. (2019)
*Caulerpa lentillifera*	In vitro synthesis	—	FDGIP	Inhibits α‐amylase (EC_50_ = 60.38 μg/mL) and α‐glucosidase (EC_50_ = 57.85 μg/mL); modulates MAPK8‐JNK1/PPARGC1A/Ghrelin/GLP‐1/CPT‐1 pathways; inhibits 3 T3‐L1 preadipocyte differentiation.	Kurniawan et al. (2024)
*Spirulina*	Enzymatic hydrolysis	Trypsin	GPNYASSER	DPP‐IV inhibition (IC_50_ = 0.358 ± 0.188 mM).	Martínez et al. (2024)
*Chlorella pyrenoidosa*	Enzymatic hydrolysis	Trypsin, pepsin	—	DPP‐IV activity reduced by 63% (pepsin hydrolysate) and 70% (trypsin hydrolysate) at 5 mg/mL.	Li et al. (2021)
Seaweed	Enzymatic hydrolysis	Alcalase papain	GGSK; ELS	Inhibits α‐amylase; IC_50_ = 2.58 ± 0.08 (GGSK), 2.62 ± 0.05 mM (ELS).	Admassu et al. (2018)
*Chlamydomonas reinhardtii*	Chemistry hydrolysis	—	—	Inhibits α‐amylase (IC_50_ =230 ± 9.5 μg/mL) and α‐glucosidase (IC₅₀ = 259 ± 8.4 μg/mL)	Siahbalaei et al. (2021)

**FIGURE 1:**
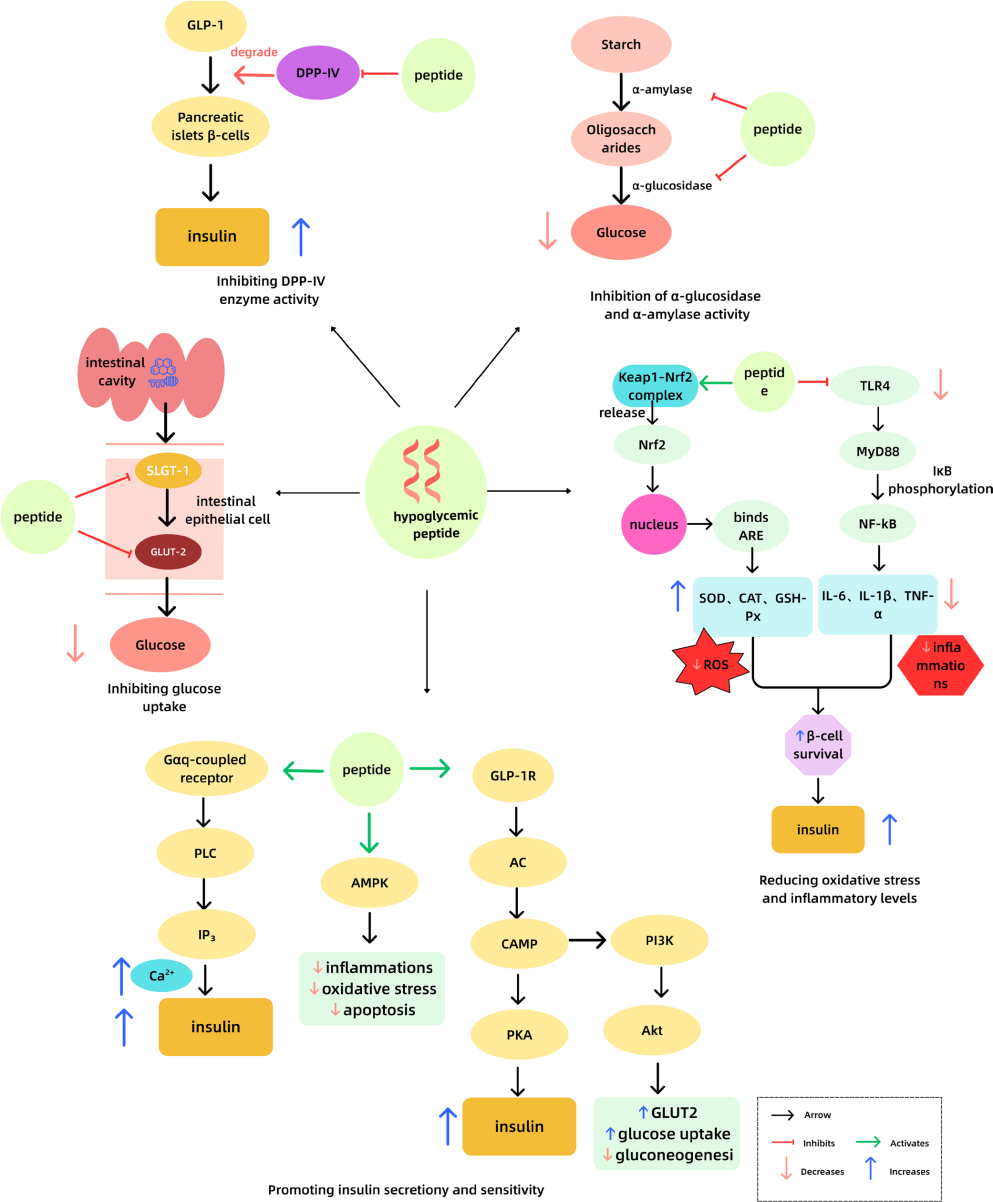
Representative mechanisms of action of hypoglycemic peptides.

Hypoglycaemic peptides can be classified according to their mechanisms of action, including enzyme‐inhibiting peptides, insulin secretion‐promoting peptides, insulin sensitivity‐enhancing peptides, antioxidant/anti‐inflammatory peptides, and glucose uptake‐modulating peptides. The specific inhibitory pathways are illustrated in Figure 1.

### 2.1 Inhibition of α‐Glucosidase and α‐Amylase Activity

Starch is initially hydrolysed by α‐amylase into oligosaccharides, which are subsequently broken down into glucose by α‐glucosidase. Therefore, inhibiting these two key digestive enzymes is considered an effective approach to controlling postprandial blood glucose levels. Hypoglycaemic peptides can slow or reduce glucose production by interfering with enzyme–substrate interactions. Depending on their mode of interaction with the enzymes, these peptides typically exhibit competitive, non‐competitive, or mixed inhibition patterns.

#### 2.1.1 Inhibiting α‐Amylase Activity

α‐Amylase comprises three domains: A, B, and C. Domains A and C constitute the primary catalytic site, while domain B is involved in substrate binding. Several key amino acid residues (e.g., Trp_58_, Trp_59_, Tyr_62_, Asp_197_, Glu_233_, His_299_, and Asp_300_) are critical for substrate or inhibitor interactions. In addition, certain allosteric sites (e.g., Asp_96_, Arg_195_, His_15_) can be occupied by bioactive peptides, inducing conformational changes that reduce enzymatic activity (Huang et al. 2024; Yin et al. 2020). Wan et al. (2023) identified multiple α‐amylase inhibitory peptides from pomfret hydrolysate, including FGNWR, CPPSPR, FNFSR, WPDAR, LSGFPR, and CPPTPR. These peptides interact with multiple domains of α‐amylase simultaneously, forming stable complexes with key residues via electrostatic interactions, hydrophobic contacts, and hydrogen bonds, thereby inducing conformational rearrangements and significantly inhibiting enzyme activity.

#### 2.1.2 Inhibiting α‐Glucosidase Activity

α‐Glucosidase inhibitory peptides typically bind rapidly to the enzyme's active site via specific amino acid residues, primarily through electrostatic interactions, hydrogen bonds, hydrophobic contacts, and van der Waals forces. This binding interferes with enzyme‐substrate complex formation and inhibits glycosidic bond hydrolysis (Kehinde et al. 2022; Dandekar et al. 2021). Xue et al. (2024) identified three α‐glucosidase inhibitory peptides—SGR, NPIY, and GPFPSI—from soybean protein. Among them, SGR exhibited the strongest inhibitory activity by forming more hydrogen bonds and interacting with a greater number of key residues, whereas NPIY and GPFPSI relied primarily on hydrogen bonds, electrostatic interactions, and hydrophobic contacts. These results are consistent with Leong and Chang (2024), who reported that low‐molecular‐weight peptides generally display higher biological activity, likely due to their simpler spatial structures facilitating more specific interactions with target enzymes or other biomolecules.

### 2.2 Inhibiting DPP‐IV Enzyme Activity

Following a meal, blood levels of GLP‐1 rise due to nutrient intake but rapidly decline because of enzymatic inactivation by DPP‐IV. Inhibiting DPP‐IV prolongs incretin half‐life, enhances insulin secretion, and suppresses glucagon release, making it an established strategy for managing type 2 diabetes (Deacon 2018). Structurally, DPP‐IV consists of an α/β hydrolase catalytic domain and a β‐helix propeller domain, forming a multi‐pocket active site capable of interacting with diverse inhibitors. Peptide‐based DPP‐IV inhibitors typically engage the S1, S2, and S3 sub‐sites via hydrogen bonds and hydrophobic interactions, with the catalytic triad residues (Ser_630_, Asp_708_, His_744_) playing a central role in inhibition (Juillerat‐Jeanneret 2013; Chai et al. 2022). Notably, inhibitory peptides comprising fewer than ten amino acid residues often display enhanced potency, likely due to better accessibility to the active site and favorable binding conformations (Ambhore et al. 2023).

Recent studies show that peptides from food and marine sources effectively inhibit DPP‐IV. For instance, Zhou et al. (2024) identified LTWR and DPF peptides from *Musculus senhousei* protease, which interact with the enzyme active pocket through hydrogen bonds and hydrophobic contacts. These findings reveal common structural features—short peptide length and presence of aromatic or hydrophobic residues—that are also prevalent in algal bioactive peptides, highlighting their potential as natural DPP‐IV inhibitors.

### 2.3 Promoting Insulin Secretion

#### 2.3.1 Via Enhancement of Glucagon‐Like Peptide −1 Activity

Glucagon‐like peptide‐1 (GLP‐1) is an incretin hormone secreted by intestinal L cells that regulates postprandial glucose by stimulating insulin secretion from pancreatic β‐cells (Zalucha 2021). GLP‐1 and its analogues act primarily through GLP‐1 receptor activation, increasing intracellular cyclic adenosine monophosphate (cAMP) levels and triggering protein kinase A (PKA)–dependent signaling pathways, thereby enhancing insulin release (Stožer et al. 2021).

In addition to promoting insulin secretion, GLP‐1 suppresses glucagon release from pancreatic α‐cells, reducing hepatic glucose production. It also delays gastric emptying and induces satiety via central nervous system signaling, collectively contributing to improved glycemic control and reduced postprandial glucose fluctuations (Jahandideh and Wu 2022; Ortizo et al. 2024). Notably, certain algal extracts have been reported to stimulate GLP‐1 secretion in vitro. Crude aqueous extracts from various brown and green algae enhance glucose‐dependent GLP‐1 release in intestinal endocrine cell models, suggesting that algal bioactive compounds, including peptides, may indirectly modulate incretin signaling (Chin et al. 2015).

#### 2.3.2 Via Enhancement of Pancreatic β‐Cell Function

β‐cell dysfunction and reduced β‐cell mass are hallmark features of type 2 diabetes mellitus (T2DM) (Inaishi and Saisho 2020). Insulin‐secreting peptides enhance β‐cell function through both direct and indirect mechanisms. Directly, these peptides activate G protein‐coupled receptor‐mediated signaling, particularly via Gαq protein, promoting phospholipase C (PLC) activation, intracellular Ca^2+^ mobilization, and subsequent insulin exocytosis (Delobel and Dalle 2021; Tran et al. 2016). For example, Tapadia et al. (2019) demonstrated that lupin hydrolysate activates PLC through Gαq protein, significantly increasing intracellular Ca^2+^ levels. This effect involves the PLC/PKC pathway, which facilitates Ca^2+^ release and mediates insulin secretion.

Indirectly, peptides support β‐cell survival and function by modulating the AMP‐activated protein kinase (AMPK) pathway, enhancing glucose sensing, improving metabolic efficiency, and reducing oxidative stress and apoptosis (Newsholme et al. 2014). Hypoglycemic peptides have been shown to regulate apoptosis‐related proteins, upregulating anti‐apoptotic Bcl‐2 and downregulating pro‐apoptotic Bax, thereby protecting β‐cells (Taneera and Saber‐Ayad 2023). In vivo studies further confirm that these peptides reduce oxidative stress, improve insulin secretion, and protect pancreatic β‐cells from diabetes‐induced damage (Olasehinde et al. 2023).

Overall, bioactive peptides exert anti‐diabetic effects not only by modulating glucose digestion and incretin signaling but also by directly enhancing β‐cell function and survival, highlighting their potential for developing algae‐derived functional peptides in diabetes management.

### 2.4 Improving Insulin Sensitivity

Hypoglycemic peptides exert insulin‐mimetic or sensitizing effects by modulating key nodes in the insulin signaling cascade. Some peptides directly interact with the insulin receptor (IR), promoting receptor autophosphorylation and activating downstream PI3K/Akt signaling. This enhances glucose uptake via increased translocation of glucose transporters (e.g., GLUT4) and promotes glycogen synthesis through regulation of glycogen synthase activity (Savova et al. 2023; Song et al. 2021).

In addition to directly activating insulin responses, hypoglycemic peptides improve insulin sensitivity by modulating intracellular signaling intermediates such as insulin receptor substrates (IRS), Akt, and AMP‐activated protein kinase (AMPK). Synergistic activation of Akt and AMPK enhances glucose uptake, suppresses gluconeogenesis, and improves metabolic flexibility in insulin‐resistant cells (Lammi et al. 2015; Wang et al. 2020; Boucher et al. 2014). For instance, Wang et al. (2020) identified a pentapeptide, LPLLR, from hydrolyzed walnut protein. At 100–200 μg/mL, LPLLR increased phosphorylation of IRS‐1, PI3K, Akt, AMPK, and GSK3β, activating insulin signaling, enhancing GS and GLUT4 expression, and promoting glycogen synthesis and glucose uptake. LPLLR also suppressed gluconeogenesis in insulin‐resistant hepatocytes by downregulating phosphoenolpyruvate carboxykinase (PEPCK) and glucose‐6‐phosphatase (G6Pase) via the IRS‐1/PI3K/Akt/FoxO1 pathway.

Furthermore, certain bioactive peptides indirectly enhance insulin responsiveness by mitigating inflammation and oxidative stress, both major contributors to insulin resistance. By reducing proinflammatory cytokine levels and improving mitochondrial function, these peptides restore insulin signaling efficiency in peripheral tissues (Hou et al. 2023).

### 2.5 Reducing Oxidative Stress and Inflammatory Levels

Oxidative stress and chronic inflammation are key contributors to pancreatic β‐cell dysfunction and insulin resistance. Hypoglycemic peptides with antioxidant and anti‐inflammatory properties can protect β‐cells and improve glycemic control by modulating redox balance and inflammatory signaling. Many peptides exert antioxidant effects by activating the nuclear factor erythroid 2‐related factor 2 (Nrf2) pathway, upregulating endogenous antioxidant enzymes such as superoxide dismutase (SOD), catalase (CAT), glutathione peroxidase (GSH‐Px), and heme oxygenase‐1 (HO‐1). Activation of Nrf2 reduces intracellular reactive oxygen species (ROS) accumulation and mitigates oxidative damage in insulin‐responsive cells (He, Xu, et al. 2023; Wang et al. 2020). Simultaneously, hypoglycemic peptides inhibit inflammatory signaling, particularly the nuclear factor kappa‐B (NF‐κB) pathway, decreasing proinflammatory cytokines such as TNF‐α, IL‐6, and IL‐1β. Suppression of pathways including TLR4/MyD88/NF‐κB helps preserve β‐cell integrity and restore insulin sensitivity (Wang, Ma, et al. 2023). Network pharmacology analyses further suggest that these anti‐inflammatory effects are closely linked to insulin signaling, PI3K/Akt, and AGE–RAGE pathways, highlighting the multi‐targeted mechanisms of peptide‐mediated glycemic regulation (Yu et al. 2024).

### 2.6 Inhibiting Glucose Uptake

Beyond systemic metabolic regulation, some hypoglycemic peptides help control blood glucose by limiting intestinal glucose absorption. They inhibit glucose transporters such as sodium‐glucose cotransporter 1 (SGLT1) and glucose transporter 2 (GLUT2), reducing postprandial glucose entry into the circulation (Abioye et al. 2022; Wicik et al. 2022). Experimental studies show that peptide hydrolysates can modulate intestinal and hepatic glucose metabolism via AMPK‐ and Akt‐related pathways, decreasing gluconeogenesis and enhancing glycogen storage. Certain peptide sequences also directly interact with SGLT1 or GLUT2, blocking glucose transport through hydrophobic and electrostatic interactions (Mojica et al. 2017; Arif et al. 2021). Overall, inhibition of glucose uptake represents a key complementary mechanism by which hypoglycemic peptides contribute to postprandial glycemic control.

## 3 Production and Characterization of Hypoglycemic Peptides

The development of hypoglycemic peptides generally follows a systematic workflow encompassing protein extraction, hydrolysis, peptide purification, structural identification, and functional evaluation. The overall research framework is illustrated in Figure S1, which outlines the sequential process from raw protein processing to bioactive peptide screening and structural confirmation. To provide a structured overview of methodological approaches at each stage, Table S1 compiles commonly employed strategies for protein extraction, hydrolysis, purification, structural characterization, and activity assessment, thereby establishing a methodological framework for hypoglycemic peptide research.

Hypoglycemic peptides are primarily derived from the enzymatic hydrolysates of food proteins. Major protein sources include dairy products, fish, legumes, cereals, and microalgae. During enzymatic hydrolysis, macromolecular proteins are cleaved into low–molecular‐weight peptide fragments, some of which have been demonstrated to exert blood glucose–regulating effects (Mora and Toldrá 2022; Yan et al. 2021). In addition, representative studies published in recent years on peptide extraction, purification, and structural characterization are summarized in Table 2. Collectively, current research in this field has focused on optimizing extraction and hydrolysis strategies, improving separation and purification efficiency, and elucidating peptide sequences and structure–activity relationships. The following sections provide a comprehensive review of these key developments.

**TABLE 2:** Recent methods on extraction, purification and identification of hypoglycemic peptides of natural origin (“—” indicates that no enzyme was used).

Source	Protein extraction methods	Enzymes used	Purification methods	Identification methods	References
*Momordica charantia* (Abbreviata variety)	Aqueous extract	—	Microfiltratio, ultrafiltration, gel chromatography, RP‐HPLC	LC–MS/MS	Yuan et al. (2008)
*Defatted camellia* seed cake	—	Alcalase	Ultrafiltration; RP‐HPLC	LC–MS/MS	Feng et al. (2021)
Sorghum spent grain	—	Purazyme; Flavourzyme	Gel chromatography	LC‐ESI‐Q‐TOF tandem mass	Garzón et al. (2022)
Goat milk	Acid precipitation	Flavourzyme	Gel chromatograph; RP‐HPLC	LC–MS/MS	Gong et al. (2020)
Fish skin ( *Merluccius productus* , *Hippoglossus stenolepis* )	Acid precipitation	Flavourzyme	Ultrafiltration	MALDI‐TOF/TOF MS/MS	Wang et al. (2015)
*Spirulina platensis*	Freeze–thaw approach; ultrasound coupled with subcritical water extraction	—	Preparative HPLC	LC–MS/MS	Hu et al. (2019)
*Spirulina*	Ultrasonication	Trypsin	Hydrophobic interaction chromatography	Nano‐LC–MS/MS	Thongcumsuk et al. (2023)
*Oreochromis niloticus*	Combinatorial ultrasound (UL)‐assisted DES‐based protein extraction	Alkaline protease	Ultrafiltration; RP‐HPLC	Amino acid profiling by amino acid analyzer; LC–MS/MS	Ortizo et al. (2024)
*Amygdalus communis L*.	Acid precipitation	Alkaline protease, neutral protease, Trypsin; Complex protease, papain	Ultrafiltration, Gel chromatography	FT‐IR, CD, HPLC, LC‐ MS	Yuan et al. (2023)
Sea buckthorn (*Hippophae rhamnoides L*.) seed meal	Water extraction	—	Macroporous resin, Ultrafiltration	LC‐ MS	Yang et al. (2024)
Rabbit meat	—	Pepsin, Flavourzyme, compound protease, Alcalase, Bromelain	Gel chromatography, RP‐HPLC	UPLC–MS/MS	Hu et al. (2024)
Pu‐erh tea	Organic solvent extraction	—	Precipitation; ultrafiltration	LC–MS/MS	Wang et al. (2024)
Bactrian camel milk	Ultrasound‐assisted enzymatic	Trypsin, α‐Chymotrypsin, Alcalase, Papain, Proteinase K	Ultrafiltration	FT‐IR, nano‐ESI‐Q Exactive‐MS	Xie et al. (2024)

### 3.1 Proteins Extraction

A common approach for protein extraction is solvent precipitation, which exploits differences in solubility or molecular weight to isolate target peptides. Traditional organic solvents, such as ethanol, methanol, acetone, and acetic acid, have been widely used in this process. However, due to potential health and environmental concerns, there is growing interest in safer and more sustainable alternatives. Green solvents, including deep eutectic solvents (DESs), bio‐based solvents, azeotropes, and dual‐solvent systems, are increasingly employed (Kamal et al. 2023). For instance, Hernández‐Corroto et al. (2020) compared two green extraction methods—pressurized liquid extraction (PLE) and assisted deep eutectic solvent extraction (AEDES)—to obtain proteins and bioactive compounds from pomegranate peels. They reported that hydrolysates from DES extracts were richer in peptides, whereas PLE extracts contained higher levels of phenolic compounds.

Additionally, physical or chemical methods are being developed to enhance protein extraction efficiency. These include ultrasound‐assisted extraction, microwave‐assisted extraction, subcritical water extraction, and enzymatic methods. Melgosa et al. (2021) applied subcritical water extraction to proteins from cod fish bones, achieving a 57.7% extraction yield and nearly complete protein recovery. Notably, the extract's inflammatory activity was reduced by 85.9% at 90°C compared with the positive control. Fan et al. (2020) combined ultrasound with subcritical water to extract peptides from *spirulina*, resulting in a protein extraction rate of 74%—about 34% higher than subcritical water extraction alone—and a much higher content of small molecule peptides (< 1000 Da) at 57.48%, comparable to those produced by enzymatic hydrolysis. Ortizo et al. (2024) compared the efficiency of protein extraction using isoelectric point precipitation, DES, and ultrasound‐assisted DES (UL‐assisted DES). They found that isoelectric point precipitation yielded 275.26 ± 6.79 mg/g of protein, DES extraction produced 293.78 ± 4.44 mg/g, and UL‐assisted DES increased the yield further to 386.37 ± 4.44 mg/g, approximately 1.4 times the rate of isoelectric point precipitation.

These findings suggest that combining these techniques can not only optimize the extraction process but also improve product quality while meeting industrial production requirements for efficiency, safety, and environmental sustainability.

### 3.2 Proteins Hydrolysis

Proteins can be hydrolysed through various methods, including chemical hydrolysis, enzymatic hydrolysis, microbial fermentation, and physical methods such as ultrasound and microwave treatment (Guo et al. 2023). Chemical hydrolysis is widely used due to its simplicity and cost‐effectiveness. However, it has significant limitations, such as difficulties in controlling the process, which can lead to changes in chemical composition (El‐Rady et al. 2023). Fermentation methods can inhibit the growth of pathogenic bacteria but are constrained by long fermentation cycles, complex product profiles, and intricate downstream extraction processes (Cruz‐Casas et al. 2021). Enzymatic hydrolysis is among the most widely used methods for producing bioactive peptides, as it enables selective cleavage of polypeptide chains at specific amino acid residues, generating defined peptide fragments while maintaining their biological activity (Valencia et al. 2015).

The efficiency of enzymatic hydrolysis and the biological activity of the resulting hydrolysates are influenced by several factors, including protein source, enzyme specificity, degree of hydrolysis, and reaction conditions (e.g., enzyme‐to‐substrate ratio, temperature, and pH). Among these variables, enzyme specificity plays a decisive role in shaping the structural characteristics of the hydrolysates, such as peptide size distribution, amino acid composition, and sequence profile (Tavano 2013). Proteases commonly employed for peptide production include animal‐derived enzymes (e.g., trypsin and pepsin), plant‐derived enzymes (e.g., papain and bromelain), and microbial proteases (e.g., neutral and alkaline proteases, as well as flavorzyme) (Naveed et al. 2021). Owing to their distinct cleavage preferences, enzyme selection critically determines the composition and functional properties of the generated peptides. For example, trypsin selectively cleaves peptide bonds at the C‐terminus of lysine and arginine residues, whereas pepsin exhibits broader cleavage specificity under acidic conditions. Alkaline proteases typically display wide substrate tolerance and multiple cleavage sites (Sujitha and Shanthi 2023; Tavano et al. 2018).

Experimental evidence further supports the importance of enzyme selection. Jia et al. (2020) reported that tryptic hydrolysis of α‐lactalbumin‐rich proteins yielded hydrolysates with the strongest DPP‐IV inhibitory activity compared with pepsin digestion, achieving an IC₅₀ value of 0.61 ± 0.036 mg/mL. Similarly, Gao et al. (2023) demonstrated that sequential dual‐enzyme hydrolysis using alkaline protease followed by pepsin–trypsin treatment produced α‐lactalbumin hydrolysates with enhanced xanthine oxidase inhibitory activity (IC_50_ = 0.28 mg/mL).

Despite its advantages, enzymatic hydrolysis faces certain limitations, including relatively slow reaction kinetics and the potential formation of undesirable by‐products. To address these challenges, enzymatic approaches are increasingly integrated with complementary techniques to improve peptide yield, enhance bioactivity, and optimize process efficiency.

### 3.3 Peptide Purification

Protein hydrolysates consist of a mixture of unhydrolyzed soluble proteins, peptides of varying molecular weights, free amino acids, and other soluble components. To enhance the biological activity of specific peptides within these hydrolysates, effective separation and purification techniques are essential. Among the most used methods are membrane separation and chromatography, both of which play a crucial role in isolating and purifying bioactive peptides for further application (Chen et al. 2025).

#### 3.3.1 Membrane Separation

Membrane separation technology leverages molecular weight and charge selectivity to effectively separate substances. It offers several advantages, including high selectivity, low energy consumption, gentle operation, environmental friendliness, and ease of scaling and upgrading (Sridhar et al. 2021). Pressure‐driven membrane operations, classified by size selectivity, include microfiltration (MF), ultrafiltration (UF), nanofiltration (NF), and reverse osmosis (RO) (Urošević and Trivunac 2020; Van Der Bruggen 2018). Ultrafiltration is particularly popular for peptide separation based on molecular weight (Ratnaningsih et al. 2021).

To enhance membrane process efficiency, enzyme membrane reactors (EMRs) have been developed, integrating membrane separation with enzymatic hydrolysis into a single operation (Hong et al. 2025). Another innovative approach is electrodialysis with ultrafiltration membranes (EDUF), which combines the size rejection capability of ultrafiltration with the charge selectivity of electrodialysis (ED) (Alavi and Ciftci 2023). For example, a study by Cournoyer et al. (2024) investigated the impact of different current conditions—continuous current (CC), pulsed electric field (PEF), and polarity reversal (PR)—on the selectivity of the EDUF process for porcine coagulant hydrolysate peptides. The study found that CC, PEF, and PR at a 10 s/1 s interval promoted the migration of predominantly low molecular weight cationic peptides, while also allowing some anionic peptides with lower molecular weights to migrate. The current conditions significantly influenced the selectivity of the migrated peptides, and peptides with potential antimicrobial activity were obtained using either the CC or PEF 10 s/1 s conditions.

Despite its extensive applications in water treatment, food processing, and the pharmaceutical industry, membrane filtration technology faces several inherent challenges. These include membrane fouling and clogging, limited selectivity, high energy and pressure demands, and a relatively short operational lifespan. Addressing these limitations requires a multifaceted approach, encompassing the development of advanced membrane materials, optimized system design, improved operational strategies, and rigorous maintenance management.

#### 3.3.2 Chromatography

Chromatographic techniques are widely used to separate and purify peptide molecules with similar chemical properties. Commonly employed methods include high‐performance liquid chromatography (HPLC), ion exchange chromatography (IEC), and gel chromatography (Debnath et al. 2025).

HPLC separates peptides based on their different partition coefficients between the stationary and mobile phases. By adjusting the composition of the mobile phase and the flow rate, HPLC can efficiently separate and purify peptides. This method is highly effective for peptides with slight differences in chemical properties (Subirats and Rosés 2025).

##### 3.3.2.1 Ion Exchange Chromatography (IEC)

IEC relies on the differences in the charges of peptides. By adjusting the pH and ionic strength of the mobile phase, peptides with different charges exhibit varying retention times on the stationary phase, leading to their separation. This method is particularly useful for peptides that differ in their charge properties (Grönberg 2018; Imiołek et al. 2025).

##### 3.3.2.2 Gel Chromatography

Also known as size‐exclusion chromatography, gel chromatography separates peptides based on differences in molecular size and shape. It uses porous fillers with specific pore sizes (e.g., dextran gels or polyacrylamide gels). This technique offers high sample recovery and operates under mild conditions, making it especially suitable for the initial separation and purification of peptides with large molecular weight differences (Zhu et al. 2025; Huang et al. 2018).

Both membrane filtration and chromatography can be used independently or in combination to achieve optimal peptide purification. The choice of purification method depends on the peptide's characteristics, purity requirements, and intended use (Musaimi and Jaradat 2024). For instance, Li, He, et al. (2023) used ultrafiltration combined with Sephadex‐G100 gel chromatography to purify and characterize a novel anti‐ACE peptide, DIGGL, derived from seaweed proteins, which exhibited a *Spirulina* of 10.32 ± 0.96 μM. Similarly, Li et al. (2024) separated anti‐ACE peptides from maize using Superdex peptide 10/300 GL and Q Sepharose High Performance anion chromatography columns, as well as Mono Q anion chromatography. This process produced three novel anti‐ACE peptides with the sequences YAEY, IIPQCS, and TIIPQ, which exhibited inhibition rates of 40.75% ± 0.72%, 21.96% ± 1.56%, and 23.56% ± 1.31%, respectively, at a concentration of 4 mg/mL.

### 3.4 Peptide Structure Analysis and Characterization

The identification of peptides encompasses the determination of their amino acid sequences, secondary and tertiary structures, and any chemical modifications. Two primary methods for amino acid sequence identification are Edman degradation and mass spectrometry (MS). Edman degradation is suitable for analyzing short peptide sequences, while MS, especially when combined with other analytical techniques, is extensively used for identifying peptide and protein sequences (Sanchez‐Avila et al. 2025; Harking et al. 2025).

For example, Radhakrishnan et al. (2024) utilized membrane filtration and reverse‐phase high‐performance liquid chromatography (RP‐HPLC) to purify antioxidant peptides from *Parmotrema perlatum*. They sequenced the peptide fractions using liquid chromatography–tandem mass spectrometry (LC–MS/MS), revealing that the peptide LSWFMVVAP exhibited the highest antioxidant activity.

Similarly, Shekoohi et al. (2024) separated protein hydrolysates from blue whiting ( *Micromesistius poutassou* ) using semi‐preparative RP‐HPLC. They identified peptides using ultra‐high‐performance liquid chromatography‐electrospray ionization mass spectrometry (UPLC‐ESI‐MS) and tandem mass spectrometry (MS/MS). Among the peptides analyzed, VPVE and IPQD demonstrated the strongest in situ DPP‐IV inhibitory activities, with IC_50_ values of 88.79 ± 5.40 and 91.12 ± 19.73 μM, respectively. Additionally, peptides MPKKE, GPAG, and MPAH were found to reduce intracellular ROS levels in HepG2 cells, with IC_50_ values of 78.59, 58.7, and 59.8 μM, respectively, playing a protective role against H_2_O_2_‐induced oxidative damage.

Peptide secondary and tertiary structures can be analyzed using various techniques, including Circular Dichroism (CD), Nuclear Magnetic Resonance (NMR), X‐ray Crystallography, Infrared Spectroscopy (IR), and Raman Spectroscopy. These methods provide detailed insights into the structural conformation of peptides, which is crucial for understanding their biological functions (Penasa et al. 2025; Wang et al. 2017).

For the analysis of chemical and post‐translational modifications, High‐Performance Liquid Chromatography (HPLC) and Mass Spectrometry (MS) are commonly employed. These techniques allow for precise identification of modifications that may influence peptide activity or stability (Long et al. 2025; Neagu et al. 2022).

These methods are often used in combination to gather comprehensive information on peptide structure. Accurate identification of peptide structures is essential for understanding their functions, studying protein interactions, and designing therapeutic drugs. This integrated approach enhances the ability to manipulate peptides for specific applications in biotechnology and medicine.

### 3.5 Peptide Hypoglycaemic Activity Determination

The assessment of hypoglycaemic peptides biological activity involves both in vitro and animal studies. In vitro assays commonly include DPP‐IV inhibitory enzyme activity, α‐glucosidase, and α‐amylase inhibitory activity to evaluate the hypoglycaemic potential of peptides (Vilcacundo et al. 2017). Additionally, various cell lines, such as pancreatic β‐cells (e.g., INS‐1, βTC‐6, MIN6), hepatocytes (HepG2), muscle cells (C2C12, L6), and adipocytes (3 T3‐L1), are used to study the effects of peptides on insulin secretion and glucose transport (Shahrestanaki et al. 2019; Yudhani et al. 2023). Animal studies are conducted using models like mice and rats, with regular monitoring of blood glucose levels and measurement of key biochemical markers in the blood, such as insulin and glycated hemoglobin (HbA1c). Advanced techniques like qPCR and Western Blot are employed to analyze the impact of peptides on the expression of critical genes (e.g., insulin, GLUT4) and to explore the signaling pathways involved, such as AMPK and PI3K/Akt. Moreover, molecular docking technology is extensively used to simulate and predict peptide interactions with target proteins (e.g., DPP‐IV, α‐amylase, α‐glucosidase), providing insights into the peptides' mechanisms of action and aiding in optimizing their structure to enhance biological activity (Han et al. 2023; Jiang et al. 2015; Ma et al. 2022).

## 4 *C. reinhardtii* (Dangeard): Prospects for Bioactive Peptide Discovery

*C. reinhardtii* is a unicellular green alga with a remarkable capacity for both photoautotrophic growth in CO_2_‐rich, light‐abundant environments and heterotrophic growth in the presence of organic carbon sources. This metabolic versatility enables it to thrive under a broad range of cultivation conditions, positioning it as an ideal bioreactor platform for the cost‐effective, sustainable, and environmentally friendly production of high‐value products such as biofuels and pharmaceuticals (Jiang et al. 2025; Tazon et al. 2026).

In addition, *C. reinhardtii* is fast‐growing, highly adaptable to various environments, and has been classified as a “Generally Recognized as Safe” (GRAS) species by the U.S. Food and Drug Administration (FDA) (Baldia et al. 2023), highlighting its strong potential for functional food development. Although *C. reinhardtii* is generally recognized as safe (GRAS) and its consumption is typically harmless to humans, the allergenicity and toxicity of its hydrolysed peptides have not yet been investigated. Existing evidence suggests that food‐derived bioactive peptides generally exhibit low allergenic and toxic potential (Perçin and Karakaya 2020; Zhang et al. 2025). Nevertheless, prior to developing *C. reinhardtii* hydrolysed peptides as functional foods or oral therapeutics, systematic safety evaluations—including cytotoxicity, allergenicity, and in vivo tolerance studies—are essential to ensure their safety and suitability for human consumption.

Multiple bioactive peptides have been identified from protein hydrolysates of *C. reinhardtii* . For instance, Su et al. (2025) screened a Salmonella‐inhibitory peptide, EWRPF, from *C. reinhardtii* protein hydrolysates, highlighting its potential antimicrobial activity. In another study, Suo et al. (2026) identified an ACE‐inhibitory peptide, IDYRY, which exhibited an IC₅₀ of 18.54 ± 5.57 μM and effectively reduced blood pressure in hypertensive rats at a dose of 20 mg/kg. Furthermore, Mao et al. (2025) discovered several monoamine oxidase A (MAO‐A) inhibitory peptides from alkaline protein hydrolysates. Among them, the peptides TEGKIPFWEGQ and GVKYGLHEVDEGATKIVQYL displayed the strongest binding affinity, interacting with MAO‐A via hydrogen bonding and hydrophobic interactions. These findings collectively demonstrate that *C. reinhardtii* –derived peptides possess diverse bioactivities, highlighting their potential as functional ingredients.

Recent studies have demonstrated that *C. reinhardtii* powder can help maintain blood glucose homeostasis and enhance immune function. In a clinical trial conducted by Zhou, Safdar, et al. (2023, 2023), 113 subjects with impaired glucose regulation (IGR) were randomly assigned to two groups: one group (*n* = 57) received daily supplementation with *C. reinhardtii* powder, while the other group (*n* = 56) received a placebo. After 90 days, the experimental group showed a significant reduction in both fasting plasma glucose (FPG) and two‐hour postprandial glucose (2hPG) levels compared to baseline (*p* < 0.05), with 2hPG also significantly lower than that of the control group (*p* < 0.01).

Further evidence of its functional benefits was provided by Fields et al. (2020), who showed that supplementation with *C. reinhardtii* mitigated weight loss and promoted recovery in a mouse model of DSS‐induced colitis, with treated mice surpassing baseline body weight by 2%, compared to an 8% loss in the control group. In a separate human study (*n* = 54), daily oral consumption of *C. reinhardtii* biomass for 1 month alleviated intestinal discomfort and diarrhea in individuals with gastrointestinal disorders, without causing significant changes to gut microbiota composition. These findings suggest that *C. reinhardtii* may support gastrointestinal health, which could, in turn, influence metabolic regulation and glycaemic control.

Taken together, *C. reinhardtii* offers multiple advantages as a novel dietary source of hypoglycaemic peptides, particularly when compared to conventional sources, including its scalability, safety profile, and multifunctional bioactivity.

Further studies suggest that *C. reinhardtii* may contain bioactive compounds with hypoglycaemic properties. For example, Khemiri et al. (2023) reported that an ethanol extract of *C. reinhardtii* inhibited α‐amylase activity by 51.0% ± 1.6% at a concentration of 5 mg/mL. However, the specific hypoglycaemic compounds responsible for this effect remain to be fully identified and characterized.

Beyond blood glucose regulation, *C. reinhardtii* has also demonstrated potential benefits for intestinal health. Fields et al. (2020) showed that supplementation with *C. reinhardtii* not only mitigated body weight loss but also promoted recovery in a mouse model of DSS‐induced colitis, with treated mice exhibiting a 2% increase in body weight compared to baseline, in contrast to an 8% weight loss observed in the control group. Additionally, in a human study (*n* = 54), daily consumption of *C. reinhardtii* biomass for 1 month effectively alleviated symptoms such as intestinal discomfort and diarrhea, without significantly altering gut microbiota composition.

**FIGURE 2:**
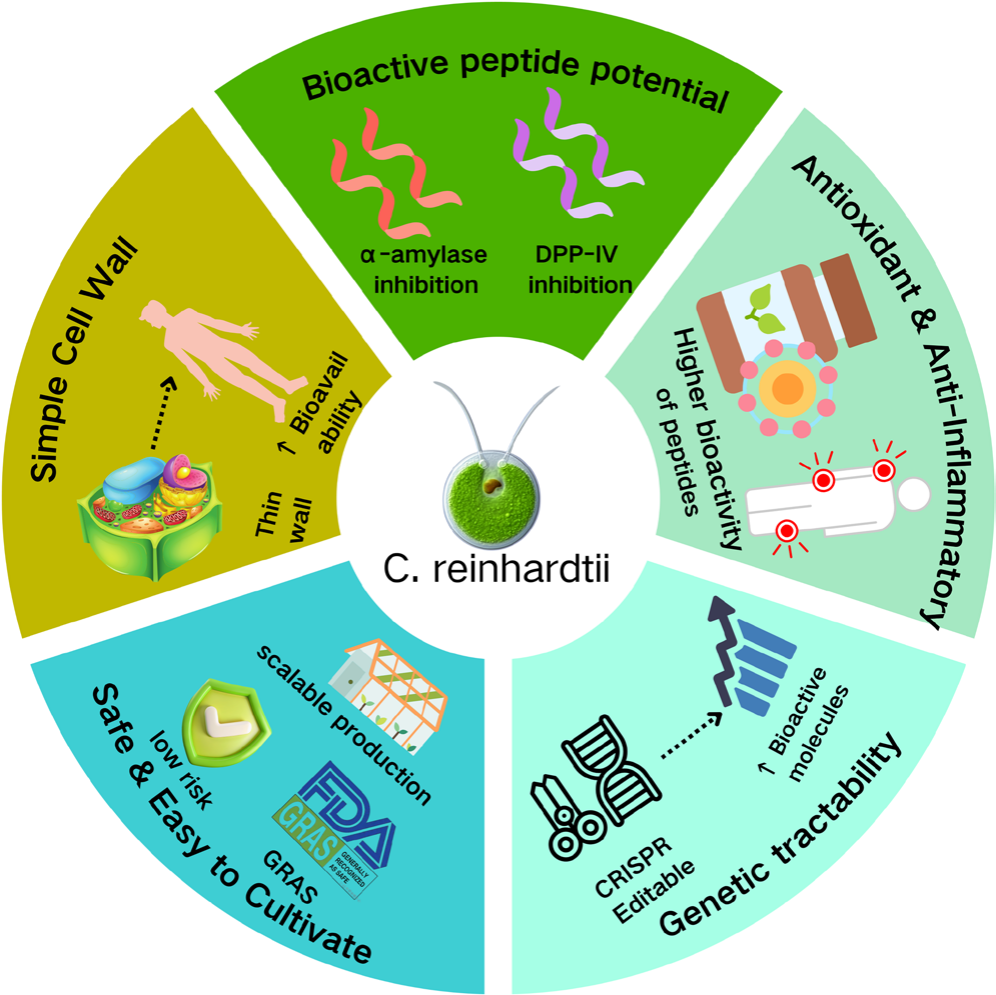
Schematic illustration of the advantages of *Chlamydomonas reinhardtii* as a source of hypoglycemic peptides.

Taken together, *C. reinhardtii* not only offers an excellent safety profile and practical applicability but also exhibits promising effects in blood glucose regulation and gut health, further underscoring its value in functional food development. As a source of hypoglycaemic peptides, *C. reinhardtii* presents several key advantages over traditional sources, as outlined below (Figure 2).

### 4.1 Potential Composition and Abundance of Hypoglycaemic‐Related Peptides in *C. reinhardtii* Proteins

The dry biomass of *C. reinhardtii* contains approximately 46.9% protein and is rich in short peptide sequences with potential biological activities. These peptides can be released as active hypoglycaemic agents during enzymatic digestion (Darwish et al. 2020). The amino acid profile of *C. reinhardtii* closely resembles that of animal proteins, containing nearly all essential amino acids. Notably, the abundance of branched‐chain amino acids such as leucine, isoleucine, and valine is believed to contribute significantly to the formation of bioactive peptides (Karami and Akbari‐Adergani 2019).

Dipeptidyl peptidase‐IV (DPP‐IV) inhibitory peptides, which play a crucial role in lowering blood glucose levels, typically comprise a mixture of hydrophobic amino acids (e.g., phenylalanine, valine, isoleucine, methionine, leucine, alanine, proline, tryptophan, glycine) and hydrophilic amino acids (e.g., arginine, histidine, threonine, lysine, glutamine, serine). The balance between hydrophilic and hydrophobic properties influences these peptides' ability to bind to the DPP‐IV active site (Ashaolu et al. 2024). Consequently, selecting appropriate enzymes during enzymatic hydrolysis can tailor the generation of peptides with specific functions, such as reducing blood glucose levels and enhancing insulin sensitivity. However, it should be emphasized that amino acid composition alone cannot be regarded as direct evidence of hypoglycaemic peptide activity, as similar profiles are commonly observed in many dietary protein sources.

By inhibiting α‐amylase and α‐glucosidase activities, these peptides may attenuate carbohydrate digestion and intestinal sugar absorption, thereby contributing to the regulation of postprandial blood glucose levels. Several studies have highlighted the significant role of certain microalgae in inhibiting α‐amylase activity, which is closely linked to their protein content, amino acid composition, and inhibitory mechanisms (Munawaroh et al. 2020). Siahbalaei et al. (2021) reported that various microalgae, including *C. reinhardtii* , *Chlorella vulgaris* , *Haematococcus*, *Umbelliferae*, and *Nestoria* spp., demonstrated inhibitory activity against both α‐amylase and α‐glucosidase. Among these, peptides extracted from *C. reinhardtii* exhibited the strongest inhibitory activity, with IC₅₀ values of 0.230 mg/mL for α‐amylase and 0.259 mg/mL for α‐glucosidase, significantly higher than those observed for other protein sources. In comparison, hydrolysates from lupin (Lupin) protein exhibited IC_50_ values of 1.66 and 1.65 mg/mL for α‐amylase and α‐glucosidase, respectively (Fadimu et al. 2022). Germinated soybean hydrolysates inhibited α‐amylase and α‐glucosidase with IC₅₀ values of 1.70 and 2.90 mg/mL, respectively (González‐Montoya et al. 2018). Small peptides (< 3 kDa) derived from milk casein hydrolysates showed an IC₅₀ of approximately 4.9 mg/mL against α‐glucosidase (Hau et al. 2025).

According to molecular docking studies reported by Siahbalaei et al. (2021), arginine, glutamic acid, and aspartic acid exhibited the strongest inhibitory potential against α‐amylase. Arginine binds directly to the enzyme's active site, whereas aspartate and glutamate likely inhibit through salt‐bridge interactions with residues Arg^398^, Arg^421^, and Asp^402^, inducing conformational changes that reduce substrate affinity. Against α‐glucosidase, arginine also displayed the strongest inhibition by forming four hydrogen bonds with Glu^296^, Asn^259^, Ile^272^, and Gly^161^. Additional stabilization arises from π‐alkyl interactions, salt bridges, and van der Waals forces, resulting in non‐competitive inhibition of the enzyme.

According to Darwish et al. (2020), *C. reinhardtii* proteins comprise 17 amino acids, with abundant glutamic acid, leucine, aspartic acid, alanine, valine, phenylalanine, lysine, arginine, and glycine. This is consistent with the observations reported by Siahbalaei et al. (2021). However, it should be noted that this field remains at an early stage, and to date, no specific hypoglycaemic peptides have been isolated or structurally characterized from *C. reinhardtii* . Overall, current evidence suggests that *C. reinhardtii* represents a promising protein source with the potential to yield hypoglycaemic peptides for blood glucose management.

### 4.2 Higher Antioxidant and Anti‐Inflammatory Activity

Oxidative stress is a key contributor to chronic inflammation and is closely associated with the development of insulin resistance and type 2 diabetes. Common reactive species involved in oxidative stress include peroxynitrite, superoxide anion, hydroxyl radicals, and nitric oxide radicals. Previous studies have shown that certain food‐derived hypoglycemic peptides also exhibit antioxidant activity, which may help alleviate oxidative stress and inflammatory damage in pancreatic β‐cells, thereby supporting β‐cell function and insulin signaling (Mijiti et al. 2025; Chiș et al. 2018).

At the level of whole biomass or crude extracts, *C. reinhardtii* has been shown to exhibit measurable antioxidant activity. Jayshree et al. (2016) reported that the methanolic extract of *C. reinhardtii* exhibited strong DPPH radical scavenging activity, with an IC_50_ value of 423.44 μg/mL. This antioxidant activity was tentatively attributed to the combined effects of chlorophyll, polyphenols, and peptide‐related components; however, the precise active constituents and their mechanisms of action remain to be clarified.

Zhou et al. (2025) reported that alkaline‐extracted *C. reinhardtii* protein (AE‐CRP) exhibited notable DPPH radical scavenging activity, with an IC₅₀ value of 9.07 mg/mL, surpassing that of several previously reported plant protein hydrolysates, such as rice bran and rice protein hydrolysates. In another study, Martínez et al. (2024) showed that *C. reinhardtii* possessed a remarkably high protein content of up to 57%, and its protein hydrolysates prepared using a broad‐spectrum endo protease from *Bacillus licheniformis* displayed exceptionally strong antioxidant capacity (9221.3 ± 111.2 μg GAE/g), significantly exceeding that of other halophilic archaea and microalgae evaluated in the same study.

Although antioxidant activity does not constitute direct evidence to produce hypoglycemic peptides, oxidative stress is widely recognized as a contributing factor to insulin resistance and type 2 diabetes. In this context, the antioxidant properties observed in *C. reinhardtii* may provide indirect physiological relevance, offering a supportive background for its investigation in metabolic health–related research rather than direct proof of hypoglycemic peptide activity.

In vivo studies further suggest that *C. reinhardtii* biomass or extracts may exert antioxidant and anti‐inflammatory effects. Xie et al. (2025) reported that oral administration of a red strain of *C. reinhardtii* significantly improved glucose metabolism in high‐fat diet–induced diabetic mice. These effects were accompanied by modulation of oxidative stress and inflammatory markers, suppression of gluconeogenic enzymes such as glucose‐6‐phosphatase (G‐6‐Pase) and phosphoenolpyruvate carboxykinase (PEPCK), and regulation of the SOCS2/JAK2/STAT5 signaling pathway. However, these observations were obtained at the level of whole biomass, and the specific bioactive components responsible for these effects, including the potential contribution of peptides, have not yet been identified.

### 4.3 Cell Wall Simplicity and Peptide Bioavailability in *C. reinhardtii*

*Spirulina*, *Chlorella*, and *C. reinhardtii* are all recognized as GRAS by regulatory authorities. Among them, *C. reinhardtii* has a distinct advantage due to its cell wall structure. Unlike the robust, multilayered cell wall of *Chlorella*, the cell walls of *Spirulina* and *C. reinhardtii* are relatively simple, consisting mainly of proteins and polysaccharides, which makes them more easily disrupted. However, the peptide‐rich matrix of *Spirulina* also contains high levels of pigments and phospholipids, potentially affecting peptide stability and absorption efficiency (Domozych et al. 2012; Darwish et al. 2020). *C. reinhardtii* has a cell wall dominated by glycoproteins rich in hydroxyproline, with minor polysaccharides (mainly arabinose and galactose, non‐cellulosic). This simpler structure is more easily disrupted by mechanical, ultrasonic, or enzymatic treatments, facilitating more efficient protein release and exposure of bioactive peptides (Sim et al. 2025). Such characteristics may reduce the energy required for gastrointestinal digestion and enhance peptide bioavailability, although further studies are needed to confirm these effects.

Preliminary in vivo evidence supports its potential. For example, the antihypertensive peptide VLVP expressed in *C. reinhardtii* was orally bioavailable and effectively reduced systolic blood pressure in rats, demonstrating stability, bioavailability, and physiological activity in the gastrointestinal tract (Ochoa‐Méndez et al. 2016). Although VLVP is not a hypoglycaemic peptide, this study illustrates the potential of *C. reinhardtii* as a platform for producing bioactive peptides and for future applications in functional foods, oral peptide therapeutics, and gut‐targeted delivery systems.

### 4.4 Genetic Engineering and Metabolic Optimization Potential

*C. reinhardtii* is widely recognized for its strong genetic tractability, making it an ideal model organism for genetic engineering and mutant strain development. Its fully sequenced genome and well‐established transformation techniques provide a solid foundation for enhancing the biosynthesis of specific functional peptides (Shamriz and Ofoghi 2016). This enables targeted expression of bioactive peptides such as DPP‐IV inhibitory peptides, GLP‐1 mimetics, and antioxidant peptides by modifying key metabolic or enzymatic pathways (Scaife et al. 2015).

Beyond its use in functional peptide expression, *C. reinhardtii* has been employed in pharmaceutical and industrial applications to produce high value‐added compounds, including essential fatty acids, carotenoids, recombinant vaccines, and therapeutic proteins (Virolainen and Chekunova 2024; Almaraz‐Delgado et al. 2014; Ochoa‐Méndez et al. 2016). It has also been utilized as a host platform for expressing functional antibodies (Baier et al. 2018). Most studies have focused on leveraging genetic and metabolic engineering to optimize the production of bioactive peptides and functional proteins.

Recent advances in chloroplast engineering have significantly improved protein expression in *C. reinhardtii* . For example, Mayfield et al. (2007) and Rosales‐Mendoza et al. (2011) demonstrated efficient chloroplast‐based expression of therapeutic proteins such as vaccines, antibodies, insulin derivatives, and hypotensive peptides. Rasala et al. (2010) successfully expressed multiple recombinant proteins—such as erythropoietin, interferon‐β, insulinogen, and VEGF—at levels up to 2%–3% of total soluble protein. Hadiatullah et al. (2020) further developed a recombinant antimicrobial peptide, 3 × Mytichitin‐CB, with an expression level of approximately 0.2%, capable of inhibiting a broad spectrum of Gram‐positive and Gram‐negative bacteria.

In the functional food context, Antonacci et al. (2021) constructed a C‐terminal psbA gene fusion system to express an antioxidant peptide in *C. reinhardtii* , yielding a transgenic strain with enhanced oxidative stress tolerance and biosensing potential. Similarly, Ochoa‐Méndez et al. (2016) engineered a chloroplast‐expressing *C. reinhardtii* strain to produce the antihypertensive peptide VLVP. Remarkably, oral administration of this transgenic strain to spontaneously hypertensive rats significantly reduced systolic blood pressure without requiring protein purification, underscoring its potential as a GRAS‐grade oral delivery platform for antihypertensive therapy.

Despite current challenges such as relatively low yields in heterologous protein expression, *C. reinhardtii* naturally contains high levels of protein and essential amino acids (Siahbalaei et al. 2021). Moreover, enzymatic hydrolysates of its proteins have shown promising bioactivities in both food and pharmaceutical applications. Therefore, enhancing the expression of hypoglycaemic peptides via genetic engineering in *C. reinhardtii* may offer a novel, efficient, and safe approach for functional food development and biotherapeutic innovation.

### 4.5 Controlled Cultivation and High Biosafety

*C. reinhardtii* , as a well‐established model microalga, has been extensively utilized in studies of photosynthetic genetics, chloroplast biology, photoreceptor structure and function, and light‐responsive behavior, owing to its well‐characterized genetic background and biological traits. It is fast‐growing, easy to cultivate, and capable of photosynthesis, with a fully defined life cycle. Notably, all three of its genomes—nuclear, chloroplast, and mitochondrial—have been fully sequenced and are amenable to genetic transformation (Virolainen and Chekunova 2024), making it a valuable platform for bioengineering applications. Furthermore, *C. reinhardtii* has a favorable biosafety profile and has been granted GRAS status, which is essential for its application in food and pharmaceutical industries (Phadnis and Prakash 2024).

From an industrial perspective, the cultivation of *C. reinhardtii* is technologically mature, allowing precise control of growth parameters and enabling efficient protein expression in artificial environments. Its rapid and controllable proliferation supports large‐scale cultivation in bioreactors, which facilitates the optimization of enzyme and functional protein production processes (Yi et al. 2026; Li et al. 2025). These characteristics not only align with the growing demand for functional hypoglycaemic foods and nutraceuticals but also contribute to the development of sustainable, eco‐friendly biomanufacturing platforms.

In the context of functional peptide development, *C. reinhardtii* demonstrates clear advantages in the enzymatic production of bioactive peptides with hypoglycaemic potential. Its proteolytic hydrolysates are rich in active peptides, exhibit high release efficiency, and show excellent biocompatibility, indicating promising application potential in the food industry. However, commercial‐scale applications are still constrained by several challenges, including high cultivation costs, suboptimal peptide yields, and the need for more efficient extraction and purification methods (Kumar et al. 2022; Soto‐Sierra et al. 2018).

To overcome these limitations and unlock its full industrial potential, future research should prioritize three key directions: (1) optimizing cultivation conditions—such as light intensity, nutrient availability, and pH—to boost algal biomass; (2) enhancing protein expression levels and improving peptide release efficiency through metabolic or genetic engineering; and (3) developing cost‐effective, scalable extraction and purification technologies. These efforts will be crucial in advancing the application of *C. reinhardtii* in functional food development and biomedical innovation.

## 5 Future Prospects and Research Challenges

*C. reinhardtii* is a unicellular green alga that does not depend on soil or arable land and can be cultivated at a large scale in closed photobioreactors (PBRs) or sealed culture systems, thereby avoiding competition with conventional agriculture (Zhu et al. 2021). As a microalgal resource generally recognized as safe (GRAS), the commercial feasibility of *C. reinhardtii* ‐derived peptides depends not only on their bioactivity but also on production yield and process scalability. Under optimized nutrient conditions, *C. reinhardtii* can achieve growth rates up to 94.6 mg/L·h, providing a robust material basis for efficient production of bioactive peptides (Zhou et al. 2025). Moreover, it is widely used as a model organism for large‐scale protein expression, and its protein extraction and downstream processing technologies are considered relatively mature (Masi et al. 2023).

Leveraging enzymatic hydrolysis and peptide extraction, *C. reinhardtii* can serve as an efficient source of bioactive peptides with antidiabetic properties. These peptides can be incorporated into functional foods or nutraceuticals, offering a promising complementary approach to conventional diabetes therapies while potentially reducing reliance on synthetic drugs. In addition, metabolic and genetic engineering strategies can further optimize peptide composition and activity, tailoring them for improved insulin‐mimetic or glucose‐lowering effects. Collectively, these features highlight the potential of *C. reinhardtii* as a sustainable, scalable, and versatile platform for the development of hypoglycemic functional foods and therapeutic applications, although systematic techno‐economic analyses remain necessary to fully assess commercial viability.

Although bioactive peptides derived from *C. reinhardtii* show considerable potential for blood glucose regulation, their translation into widespread use for diabetes management faces several critical challenges. From a production standpoint, large‐scale generation of highly active peptides remains technically demanding. Optimizing enzymatic hydrolysis requires careful selection of enzyme type, hydrolysis duration, and dosage, as well as precise control over the degree of hydrolysis to prevent structural degradation of bioactive peptides or formation of non‐specific fragments. In addition, peptides of different molecular weights exhibit varied inhibitory effects on hypoglycaemic targets, such as α‐amylase, α‐glucosidase, DPP‐4, and GLP‐1, which requires the use of advanced fractionation techniques, including membrane separation and chromatography, to enrich target peptides. Achieving this at an industrial scale remains challenging.

Clinical validation is another essential step for practical application. Systematic human intervention studies are needed to evaluate toxicity, allergenicity, and gastrointestinal stability, as well as the hypoglycemic efficacy of *C. reinhardtii* ‐derived peptides in healthy individuals, people with impaired glucose tolerance, and type 2 diabetes patients. Key endpoints should include postprandial glucose levels, area under the glucose curve (AUC), insulin sensitivity, bioavailability, and potential adverse effects. To date, most evidence is limited to in vitro enzyme inhibition assays and animal models, with clinical data largely lacking.

Moreover, regulatory approval for use in functional foods or nutritional supplements requires comprehensive safety assessments, including acute and sub‐chronic toxicity testing, evaluation of mutagenicity and allergenic potential, and long‐term intake safety studies. Regulatory agencies across different regions set high standards for data completeness and safety documentation.

In summary, realizing the full potential of *C. reinhardtii* ‐derived peptides in diabetes management will require multidisciplinary efforts that integrate bioprocess engineering, food science, and clinical nutrition research, facilitating their translation from laboratory studies to functional food and nutraceutical applications.

## 6 Conclusion

This review highlights the promise of *C. reinhardtii* ‐derived bioactive peptides in diabetes management, particularly for type 2 diabetes mellitus (T2DM). Compared to conventional treatments, these peptides offer a safe, sustainable, and potentially more tolerable alternative due to their hypoglycaemic effects and ease of cultivation. The enzymatic hydrolysis of *C. reinhardtii* proteins has demonstrated the ability to produce bioactive peptides with blood sugar‐lowering properties, making them a compelling candidate for functional food and nutraceutical applications.

However, to fully harness the potential of *C. reinhardtii* ‐derived peptides, further research is required to optimize large‐scale production, improve extraction efficiency, and validate their efficacy through rigorous clinical trials. Addressing these challenges will be key to translating this emerging field into real‐world applications. With ongoing advancements in biotechnology, peptide engineering, and clinical nutrition, *C. reinhardtii* may pave the way for next‐generation functional foods and peptide‐based therapeutics for diabetes management.

## Author Contributions

**Xiaoyan Sun:** data curation. **Jing Huang:** investigation. **Keying Su:** data curation. **Qian Li:** conceptualization, writing – original draft, formal analysis. **Lai‐Hoong Cheng:** supervision, writing – review and editing. **Chunmin Yang:** investigation. **Saiyi Zhong:** project administration, writing – review and editing.

## Funding

This research were supported by the Industry University Co‐operation Collaborative Education Project, Ministry of Education, China (no. 231103880091918), and Guangzhou College of Technology and Business Research Project (grant no. KYYB202432) and 2024 Research Capability Enhancement Project of Guangdong Key Academic Discipline Development: Research on Green and Precise Mitigation Technology for Bongkrekic Acid (2024ZDJS089).

## Conflicts of Interest

The authors declare no conflicts of interest.

## Supporting information


**Figure S1:** Preparation workflow of bioactive peptides.

**Table S1:** Overview of methods for protein and peptide extraction, purification, identification, and bioactivity evaluation.

## Data Availability

No new data were created or analyzed in this study. Data sharing is not applicable to this article.

## References

[fsn371790-bib-0001] Abioye, R. O. , I. U. Okagu , and C. C. Udenigwe . 2022. “Targeting Glucose Transport Proteins for Diabetes Management: Regulatory Roles of Food‐Derived Compounds.” Journal of Agricultural and Food Chemistry 70, no. 17: 5284–5290. 10.1021/acs.jafc.2c00817. 35439410

[fsn371790-bib-0002] Accili, D. , Z. Deng , and Q. Liu . 2025. “Insulin Resistance in Type 2 Diabetes Mellitus.” Nature Reviews Endocrinology 21, no. 7: 413–426. 10.1038/s41574-025-01114-y. 40247011

[fsn371790-bib-0003] Admassu, H. , M. A. A. Gasmalla , R. Yang , and W. Zhao . 2018. “Identification of Bioactive Peptides With α‐Amylase Inhibitory Potential From Enzymatic Protein Hydrolysates of Red Seaweed (*Porphyra spp*.).” Journal of Agricultural and Food Chemistry 66, no. 19: 4872–4882. 10.1021/acs.jafc.8b00960. 29667406

[fsn371790-bib-0004] Aktas, G. 2025. “Exploring the Link: Hemogram‐Derived Markers in Type 2 Diabetes Mellitus and Its Complications.” World Journal of Diabetes 16, no. 7: 105233. 10.4239/wjd.v16.i7.105233. 40697587 PMC12278093

[fsn371790-bib-0005] Alavi, F. , and O. N. Ciftci . 2023. “Purification and Fractionation of Bioactive Peptides Through Membrane Filtration: A Critical and Application Review.” Trends in Food Science & Technology 131: 118–128. 10.1016/j.tifs.2022.11.024.

[fsn371790-bib-0006] Almaraz‐Delgado, A. L. , J. Flores‐Uribe , V. H. Pérez‐España , E. Salgado‐Manjarrez , and J. A. Badillo‐Corona . 2014. “Production of Therapeutic Proteins in the Chloroplast of *Chlamydomonas reinhardtii* .” AMB Express 4, no. 1: 1–9. 10.1186/s13568-014-0057-4. 25136510 PMC4131161

[fsn371790-bib-0007] Ambhore, J. P. , P. R. Laddha , A. Nandedkar , et al. 2023. “Medicinal Chemistry of Non‐Peptidomimetic Dipeptidyl Peptidase IV (DPP IV) Inhibitors for Treatment of Type 2 Diabetes Mellitus: Insights on Recent Development.” Journal of Molecular Structure 1284: 135249. 10.1016/j.molstruc.2023.135249.

[fsn371790-bib-0008] Antonacci, A. , I. Bertalan , M. T. Giardi , et al. 2021. “Enhancing Resistance of *Chlamydomonas reinhardtii* to Oxidative Stress Fusing Constructs of Heterologous Antioxidant Peptides Into D1 Protein.” Algal Research 54: 102184. 10.1016/j.algal.2021.102184.

[fsn371790-bib-0009] Arif, R. , S. Ahmad , G. Mustafa , et al. 2021. “Molecular Docking and Simulation Studies of Antidiabetic Agents Devised From Hypoglycemic Polypeptide‐P of *Momordica charantia* .” BioMed Research International 2021: 5561129. 10.1155/2021/5561129. 34589547 PMC8476269

[fsn371790-bib-0010] Ashaolu, T. J. , J. Olatunji , A. Karaca , et al. 2024. “Anti‐Obesity and Anti‐Diabetic Bioactive Peptides: A Comprehensive Review of Their Sources, Properties, and Techno‐Functional Challenges.” Food Research International 187: 114427. 10.1016/j.foodres.2024.114427. 38763677

[fsn371790-bib-0011] Baier, T. , D. Kros , R. C. Feiner , K. J. Lauersen , K. M. Müller , and O. Kruse . 2018. “Engineered Fusion Proteins for Efficient Protein Secretion and Purification of a Human Growth Factor From the Green Microalga *Chlamydomonas reinhardtii* .” ACS Synthetic Biology 7, no. 11: 2547–2557. 10.1021/acssynbio.8b00226. 30296377

[fsn371790-bib-0012] Baldia, A. , D. Rajput , A. Kumar , A. Pandey , and K. K. Dubey . 2023. “Engineering Microalgae as the Next‐Generation Food.” Systems Microbiology and Biomanufacturing 3, no. 1: 166–178. 10.1007/s43393-022-00144-1.

[fsn371790-bib-0013] Bhandari, D. , S. Rafiq , Y. Gat , P. Gat , R. Waghmare , and V. Kumar . 2020. “A Review on Bioactive Peptides: Physiological Functions, Bioavailability and Safety.” International Journal of Peptide Research and Therapeutics 26, no. 1: 139–150. 10.1007/s10989-019-09823-5.

[fsn371790-bib-0014] Bonora, B. M. , A. Avogaro , and G. P. Fadini . 2020. “Extraglycemic Effects of SGLT2 Inhibitors: A Review of the Evidence.” Diabetes, Metabolic Syndrome and Obesity 13: 161–174. 10.2147/DMSO.S233538. PMC6982447 32021362

[fsn371790-bib-0015] Boucher, J. , A. Kleinridders , and C. R. Kahn . 2014. “Insulin Receptor Signaling in Normal and Insulin‐Resistant States.” Cold Spring Harbor Perspectives in Biology 6, no. 1: a009191. 10.1101/cshperspect.a009191. 24384568 PMC3941218

[fsn371790-bib-0016] Cai, L. , S. Wu , C. Jia , C. Cui , and D. Sun‐Waterhouse . 2023. “Active Peptides With Hypoglycemic Effect Obtained From Hemp ( *Cannabis sativa* L.) Protein Through Identification, Molecular Docking, and Virtual Screening.” Food Chemistry 429: 136912. 10.1016/j.foodchem.2023.136912. 37480780

[fsn371790-bib-0017] Campbell, J. E. , and C. B. Newgard . 2021. “Mechanisms Controlling Pancreatic Islet Cell Function in Insulin Secretion.” Nature Reviews Molecular Cell Biology 22, no. 2: 142–158. 10.1038/s41580-020-00317-7. 33398164 PMC8115730

[fsn371790-bib-0018] Ceriello, A. , F. Prattichizzo , M. Phillip , et al. 2021. “Glycaemic Management in Diabetes: Old and New Approaches.” Lancet Diabetes & Endocrinology 10, no. 1: 75–84. 10.1016/s2213-8587(21)00245-x. 34793722

[fsn371790-bib-0019] Chai, T. , C. C. Wong , M. Z. Sabri , T. T. Chai , and F. C. Wong . 2022. “Seafood Paramyosins as Sources of Anti‐Angiotensin‐Converting‐Enzyme and Anti‐Dipeptidyl‐Peptidase Peptides After Gastrointestinal Digestion: A Cheminformatic Investigation.” Molecules 27, no. 12: 3864. 10.3390/molecules27123864. 35744987 PMC9229108

[fsn371790-bib-0020] Chen, G. , Y. Wan , and R. Ghosh . 2025. “Bioseparation Using Membrane Chromatography: Innovations, and Challenges.” Journal of Chromatography A 1744: 465733. 10.1016/j.chroma.2025.465733. 39893917

[fsn371790-bib-0022] Chin, Y. X. , P. E. Lim , C. A. Maggs , S. M. Phang , Y. Sharifuddin , and B. D. Green . 2015. “Anti‐Diabetic Potential of Selected Malaysian Seaweeds.” Journal of Applied Phycology 27, no. 5: 2137–2148. 10.1007/s10811-014-0462-8.

[fsn371790-bib-0023] Chiș, I. C. , A. Clichici , R. Simedrea , et al. 2018. “The Effects of a New Chromenyl‐Methylenethiazolidine‐2,4‐Dione in Alleviating Oxidative Stress in a Rat Model of Streptozotocin Induced Diabetes.” Studia Universitatis Babes‐Bolyai, Chemia 63: 103–112. 10.24193/subbchem.2018.4.08.

[fsn371790-bib-0024] Cournoyer, A. , G. Daigle , J. Thibodeau , V. Perreault , and L. Bazinet . 2024. “Effects of Applied Current Modes on the Migration Selectivity of Peptides From a Porcine Cruor Hydrolysate During Electrodialysis With Ultrafiltration Membrane.” Separation and Purification Technology 338: 126280. 10.1016/j.seppur.2024.126280.

[fsn371790-bib-0025] Cruz‐Casas, D. E. , C. N. Aguilar , J. A. Ascacio‐Valdés , R. Rodríguez‐Herrera , M. L. Chávez‐González , and A. C. Flores‐Gallegos . 2021. “Enzymatic Hydrolysis and Microbial Fermentation: The Most Favorable Biotechnological Methods for the Release of Bioactive Peptides.” Food Chemistry: Molecular Sciences 3: 100047. 10.1016/j.fochms.2021.100047. 35415659 PMC8991988

[fsn371790-bib-0026] Dandekar, P. , S. Ramkumar , and A. RaviKumar . 2021. “Structure–Activity Relationships of Pancreatic α‐Amylase and α‐Glucosidase as Antidiabetic Targets.” Studies in Natural Products Chemistry 70: 381–410. 10.1016/B978-0-12-819489-8.00014-4.

[fsn371790-bib-0027] Darwish, R. , M. A. Gedi , P. Akepach , H. Assaye , A. S. Zaky , and D. A. Gray . 2020. “ *Chlamydomonas reinhardtii* Is a Potential Food Supplement With the Capacity to Outperform *Chlorella* and *Spirulina* .” Applied Sciences 10, no. 19: 6736. 10.3390/app10196736.

[fsn371790-bib-0028] De Castro, R. J. S. , and H. H. Sato . 2015. “Biologically Active Peptides: Processes for Their Generation, Purification and Identification and Applications as Natural Additives in the Food and Pharmaceutical Industries.” Food Research International 74: 185–198. 10.1016/j.foodres.2015.05.013. 28411983

[fsn371790-bib-0029] Deacon, C. F. 2018. “Peptide Degradation and the Role of DPP‐4 Inhibitors in the Treatment of Type 2 Diabetes.” Peptides 100: 150–157. 10.1016/j.peptides.2017.10.011. 29412814

[fsn371790-bib-0030] Debnath, S. , M. Das , S. Mondal , et al. 2025. “Advances in Chromatography: Contemporary Techniques and Applications.” Essential Chem 2, no. 1: 1–27. 10.1080/28378083.2025.2466624.40248686

[fsn371790-bib-0031] Delobel, M. , and S. Dalle . 2021. “G‐Protein–Coupled Receptors Controlling Pancreatic β‐Cell Functional Mass for the Treatment of Type 2 Diabetes.” Current Opinion in Endocrine and Metabolic Research 16: 113–118. 10.1016/j.coemr.2020.09.010.

[fsn371790-bib-0032] Domozych, D. S. , M. Ciancia , J. Fangel , et al. 2012. “The Cell Walls of Green Algae: A Journey Through Evolution and Diversity.” Frontiers in Plant Science 3: 82. 10.3389/fpls.2012.00082. 22639667 PMC3355577

[fsn371790-bib-0033] El‐Rady, T. K. A. , A. M. Tahoun , M. Abdin , et al. 2023. “Effect of Different Hydrolysis Methods on Composition and Functional Properties of Fish Protein Hydrolysate Obtained From Bigeye Tuna Waste.” International Journal of Food Science & Technology 58, no. 12: 6552–6562. 10.1111/ijfs.16769.

[fsn371790-bib-0034] Fadimu, G. J. , A. Farahnaky , H. Gill , O. A. Olalere , C. Y. Gan , and T. Truong . 2022. “In‐Silico Analysis and Antidiabetic Effect of α‐Amylase and α‐Glucosidase Inhibitory Peptides From Lupin Protein Hydrolysate: Enzyme‐Peptide Interaction Study Using Molecular Docking Approach.” Food 11, no. 21: 3375. 10.3390/foods11213375. PMC9656729 36359988

[fsn371790-bib-0035] Fan, X. , S. Hu , K. Wang , R. Yang , and X. Zhang . 2020. “Coupling of Ultrasound and Subcritical Water for Peptides Production From *Spirulina platensis* .” Food and Bioproducts Processing 121: 105–112. 10.1016/j.fbp.2020.01.012.

[fsn371790-bib-0036] Feng, J. , Y. L. Ma , P. Sun , et al. 2021. “Purification and Characterisation of α‐Glucosidase Inhibitory Peptides From Defatted Camellia Seed Cake.” International Journal of Food Science & Technology 56, no. 1: 138–147. 10.1111/ijfs.14613.

[fsn371790-bib-0037] Fields, F. J. , F. Lejzerowicz , D. Schroeder , et al. 2020. “Effects of the Microalgae *Chlamydomonas* on Gastrointestinal Health.” Journal of Functional Foods 65: 103738. 10.1016/j.jff.2019.103738.

[fsn371790-bib-0038] Forouhi, N. G. , and N. J. Wareham . 2018. “Epidemiology of Diabetes.” Medicine 47, no. 1: 22–27. 10.1016/j.mpmed.2018.10.004. PMC4282306 25568613

[fsn371790-bib-0039] Gajjar, A. , A. Gajjar , A. K. Raju , et al. 2025. “SGLT2 Inhibitors and GLP‐1 Receptor Agonists in Cardiovascular–Kidney–Metabolic Syndrome.” Biomedicine 13, no. 8: 1924. 10.3390/biomedicines13081924. PMC12383900 40868177

[fsn371790-bib-0040] Gao, Y. F. , M. Q. Liu , Z. H. Li , et al. 2023. “Purification and Identification of Xanthine Oxidase Inhibitory Peptides From Enzymatic Hydrolysate of α‐Lactalbumin and Bovine Colostrum Casein.” Food Research International 169: 112882. 10.1016/j.foodres.2023.112882. 37254330

[fsn371790-bib-0041] Garzón, A. G. , F. F. Veras , A. Brandelli , and S. R. Drago . 2022. “Purification, Identification and in Silico Studies of Antioxidant, Antidiabetogenic and Antibacterial Peptides Obtained From sorghum Spent Grain Hydrolysate.” LWT 153: 112414. 10.1016/j.lwt.2021.112414.

[fsn371790-bib-0042] Gedif, H. , and J. Tkaczewska . 2024. “Sourcing, Use of Biopeptides, and Active Protein Hydrolysates as a Positive Response to Green Politics in the World—Current State and Challenges: A Review.” Food and Bioprocess Technology 17, no. 12: 4450–4472. 10.1007/s11947-024-03382-4.

[fsn371790-bib-0044] Gong, H. , J. Gao , Y. Wang , et al. 2020. “Identification of Novel Peptides From Goat Milk Casein That Ameliorate High‐Glucose‐Induced Insulin Resistance in HepG2 Cells.” Journal of Dairy Science 103, no. 6: 4907–4918. 10.3168/jds.2019-17513. 32253041

[fsn371790-bib-0045] González‐Montoya, M. , B. Hernández‐Ledesma , R. Mora‐Escobedo , and C. Martínez‐Villaluenga . 2018. “Bioactive Peptides From Germinated Soybean With Anti‐Diabetic Potential by Inhibition of Dipeptidyl Peptidase‐IV, α‐Amylase, and α‐Glucosidase Enzymes.” International Journal of Molecular Sciences 19, no. 10: 2883. 10.3390/ijms19102883. 30249015 PMC6213256

[fsn371790-bib-0046] Grönberg, A. 2018. “Ion Exchange Chromatography.” In Elsevier eBooks, 379–399. 10.1016/b978-0-08-100623-8.00018-9.

[fsn371790-bib-0047] Guo, Q. , P. Chen , and X. Chen . 2023. “Bioactive Peptides Derived From Fermented Foods: Preparation and Biological Activities.” Journal of Functional Foods 101: 105422. 10.1016/j.jff.2023.105422.

[fsn371790-bib-0048] Hadiatullah, H. , H. Wang , Y. X. Liu , and Z. C. Fan . 2020. “ *Chlamydomonas reinhardtii* ‐Derived Multimer Mytichitin‐CB Possesses Potent Antibacterial Properties.” Process Biochemistry 96: 21–29. 10.1016/j.procbio.2020.05.010.

[fsn371790-bib-0049] Han, C. , X. Kong , X. Xia , et al. 2023. “Effects of Ginseng Peptides on the Hypoglycemic Activity and Gut Microbiota of a Type 2 Diabetes Mellitus Mice Model.” Journal of Functional Foods 111: 105897. 10.1016/j.jff.2023.105897.

[fsn371790-bib-0050] Harking, F. , U. H. Guzman , J. Kraegenbring , et al. 2025. “Enhancing Tandem MS Sensitivity and Peptide Identification via Ion Preaccumulation in an orbitRap Mass Spectrometer.” Journal of Proteome Research 24, no. 8: 4292–4299. 10.1021/acs.jproteome.5c00186. 40583720 PMC12322952

[fsn371790-bib-0052] Hau, E. H. , W. Huang , L. Huang , et al. 2025. “Antidiabetic Potential of Buffalo Milk Casein Hydrolysates Through Enzymatic Hydrolysis and Bioactive Peptide Identification as α‐Glucosidase Inhibitors.” Food Chemistry 493, no. Pt 3: 145780. 10.1016/j.foodchem.2025.145780. 40829443

[fsn371790-bib-0054] He, S. , Z. Xu , J. Li , Y. Guo , Q. Lin , and H. Jin . 2023. “Peptides From *Harpadon nehereus* Protect Against Hyperglycemia‐Induced HepG2 via Oxidative Stress and Glycolipid Metabolism Regulation.” Journal of Functional Foods 108: 105723. 10.1016/j.jff.2023.105723.

[fsn371790-bib-0055] Hernández‐Corroto, E. , M. Plaza , M. L. Marina , and M. C. García . 2020. “Sustainable Extraction of Proteins and Bioactive Substances From Pomegranate Peel ( *Punica granatum* L.) Using Pressurized Liquids and Deep Eutectic Solvents.” Innovative Food Science & Emerging Technologies 60: 102314. 10.1016/j.ifset.2020.102314.

[fsn371790-bib-0056] Hong, J. , Y. Chang , H. Feng , L. Jiang , F. Wu , and Z. He . 2025. “A New Technique for Antioxidant Walnut Peptide Preparation Directly From Walnut Cake: Enzymatic Preparation Process Optimization Coupled With Enzyme Membrane Reactor and Kinetic Analysis.” Food Chemistry 475: 143368. 10.1016/j.foodchem.2025.143368. 39970570

[fsn371790-bib-0057] Hou, W. , F. Zhao , L. Fang , et al. 2023. “Walnut‐Derived Peptides Promote Autophagy via the Activation of AMPK/mTOR/ULK1 Pathway to Ameliorate Hyperglycemia in Type 2 Diabetic Mice.” Journal of Agricultural and Food Chemistry 71, no. 8: 3751–3765. 10.1021/acs.jafc.2c07112. 36802594

[fsn371790-bib-0058] Hu, H. , J. Li , F. Chen , et al. 2024. “Isolation and Characterization of DPP‐IV Inhibitory Peptide From Rabbit Meat Hydrolysate: Mechanism, Gastrointestinal Resistance, and Hypoglycemic Effects of Leucyl‐Leucine (LL).” Food Bioscience 61: 104592. 10.1016/j.fbio.2024.104592.

[fsn371790-bib-0059] Hu, S. , X. Fan , P. Qi , and X. Zhang . 2019. “Identification of Anti‐Diabetes Peptides From *Spirulina platensis* .” Journal of Functional Foods 56: 333–341. 10.1016/j.jff.2019.03.024.

[fsn371790-bib-0060] Huang, T. Y. , L. M. Chi , and K. Y. Chien . 2018. “Size‐Exclusion Chromatography Using Reverse‐Phase Columns for Protein Separation.” Journal of Chromatography A 1571: 201–212. 10.1016/j.chroma.2018.08.020. 30146374

[fsn371790-bib-0061] Huang, Y. , S. J. Richardson , C. S. Brennan , and S. Kasapis . 2024. “Mechanistic Insights Into α‐Amylase Inhibition, Binding Affinity and Structural Changes Upon Interaction With Gallic Acid.” Food Hydrocolloids 148: 109467. 10.1016/j.foodhyd.2023.109467.

[fsn371790-bib-0062] Imiołek, M. , S. Fekete , S. Rudaz , and D. Guillarme . 2025. “Ion Exchange Chromatography of Biotherapeutics: Fundamental Principles and Advanced Approaches.” Journal of Chromatography A 1742: 465672. 10.1016/j.chroma.2025.465672. 39805233

[fsn371790-bib-0063] Inaishi, J. , and Y. Saisho . 2020. “Beta‐Cell Mass in Obesity and Type 2 Diabetes, and Its Relation to Pancreas Fat: A mini‐Review.” Nutrients 12, no. 12: 3846. 10.3390/nu12123846. 33339276 PMC7766247

[fsn371790-bib-0064] Jahandideh, F. , and J. Wu . 2022. “A Review on Mechanisms of Action of Bioactive Peptides Against Glucose Intolerance and Insulin Resistance.” Food Science and Human Wellness 11, no. 6: 1441–1454. 10.1016/j.fshw.2022.06.001.

[fsn371790-bib-0065] Jayshree, A. , S. Jayashree , and N. Thangaraju . 2016. “ *Chlorella* Vulgaris and *Chlamydomonas reinhardtii* : Effective Antioxidant, Antibacterial and Anticancer Mediators.” Indian Journal of Pharmaceutical Sciences 78, no. 5: 575–581.

[fsn371790-bib-0066] Jia, C. L. , N. Hussain , J. Ujiroghene , et al. 2020. “Generation and Characterization of Dipeptidyl Peptidase‐IV Inhibitory Peptides From Trypsin‐Hydrolyzed α‐Lactalbumin‐Rich Whey Proteins.” Food Chemistry 318: 126333. 10.1016/j.foodchem.2020.126333. 32151919

[fsn371790-bib-0067] Jiang, N. , S. Zhang , J. Zhu , J. Shang , and X. Gao . 2015. “Hypoglycemic, Hypolipidemic and Antioxidant Effects of Peptides From Red Deer Antlers in Streptozotocin‐Induced Diabetic Mice.” Tohoku Journal of Experimental Medicine 236, no. 1: 71–79. 10.1620/tjem.236.71. 25985857

[fsn371790-bib-0068] Jiang, S. , Q. Liu , T. Gao , et al. 2025. “3D‐Printable Emulsion Gels Structured via Depletion Attraction From *Chlamydomonas reinhardtii* Biomass.” Food Research International 221, no. Pt 1: 117286. 10.1016/j.foodres.2025.117286. 41606871

[fsn371790-bib-0069] Juillerat‐Jeanneret, L. 2013. “Dipeptidyl Peptidase IV and Its Inhibitors: Therapeutics for Type 2 Diabetes and What Else?” Journal of Medicinal Chemistry 57, no. 6: 2197–2212. 10.1021/jm400658e. 24099035

[fsn371790-bib-0070] Kamal, H. , A. Ali , S. Manickam , and C. F. le . 2023. “Impact of Cavitation on the Structure and Functional Quality of Extracted Protein From Food Sources – An Overview.” Food Chemistry 407: 135071. 10.1016/j.foodchem.2022.135071. 36493478

[fsn371790-bib-0071] Karami, Z. , and B. Akbari‐Adergani . 2019. “Bioactive Food Derived Peptides: A Review on Correlation Between Structure of Bioactive Peptides and Their Functional Properties.” Journal of Food Science and Technology 56, no. 2: 535–547. 10.1007/s13197-018-3549-4. 30906011 PMC6400753

[fsn371790-bib-0072] Kehinde, O. A. , D. B. I. Damilare , A. Ogunlana , et al. 2022. “Inhibitors of α‐Glucosidase and Angiotensin‐Converting Enzyme in the Treatment of Type 2 Diabetes and Its Complications: A Review on in Silico Approach.” Pharmaceutical and Biomedical Research 8, no. 4: 237–258. 10.32598/pbr.8.4.1052.1.

[fsn371790-bib-0073] Khakhariya, R. , A. A. Sakure , R. Maurya , et al. 2023. “A Comparative Study of Fermented Buffalo and Camel Milk With Anti‐Inflammatory, ACE‐Inhibitory and Anti‐Diabetic Properties and Release of Bioactive Peptides With Molecular Interactions: In Vitro, in Silico and Molecular Study.” Food Bioscience 52: 102373. 10.1016/j.fbio.2023.102373.

[fsn371790-bib-0074] Khemiri, S. , N. Khelifi , C. Messaoud , and I. Smaali . 2023. “Bioprospecting of Microalgae for a Potential Use as Enzyme Inhibitors, Anti‐Ageing and Prebiotic Agents.” Biocatalysis and Agricultural Biotechnology 51: 102759. 10.1016/j.bcab.2023.102759.

[fsn371790-bib-0075] Khunti, K. , F. Zaccardi , A. Amod , et al. 2024. “Glycaemic Control Is Still Central in the Hierarchy of Priorities in Type 2 Diabetes Management.” Diabetologia 68, no. 1: 17–28. 10.1007/s00125-024-06254-w. 39155282 PMC11663178

[fsn371790-bib-0076] Kumar, R. , A. S. Hegde , K. Sharma , P. Parmar , and V. Srivatsan . 2022. “Microalgae as a Sustainable Source of Edible Proteins and Bioactive Peptides – Current Trends and Future Prospects.” Food Research International 157: 111338. 10.1016/j.foodres.2022.111338. 35761613

[fsn371790-bib-0077] Kurniawan, R. , N. A. Taslim , H. Hardinsyah , et al. 2024. “Pharmacoinformatics and Cellular Studies of Algal Peptides as Functional Molecules to Modulate Type‐2 Diabetes Markers.” Future Foods 9: 100354. 10.1016/j.fufo.2024.100354.

[fsn371790-bib-0078] Lammi, C. , C. Zanoni , and A. Arnoldi . 2015. “Three Peptides From Soy Glycinin Modulate Glucose Metabolism in Human Hepatic HepG2 Cells.” International Journal of Molecular Sciences 16, no. 11: 27362–27370. 10.3390/ijms161126029. 26580610 PMC4661887

[fsn371790-bib-0079] Lebovitz, H. E. 2019. “Thiazolidinediones: The Forgotten Diabetes Medications.” Current Diabetes Reports 19, no. 12: 1–13. 10.1007/s11892-019-1270-y. 31776781 PMC6881429

[fsn371790-bib-0080] Leong, Y. K. , and J. S. Chang . 2024. “Proteins and Bioactive Peptides From Algae: Insights Into Antioxidant, Anti‐Hypertensive, Anti‐Diabetic and Anti‐cancer Activities.” Trends in Food Science & Technology 145: 104352. 10.1016/j.tifs.2024.104352.

[fsn371790-bib-0081] Li, G. , X. Liu , Z. Miao , and X. Zheng . 2024. “Purification and Structural Characterization of Three Novel Anti‐Adhesive Peptides Against *Helicobacter pylori* From Corn Gluten Meal.” Journal of Functional Foods 112: 105992. 10.1016/j.jff.2023.105992.

[fsn371790-bib-0082] Li, W. , X. Fu , T. Zhang , H. Li , T. Chen , and X. Liu . 2023. “Isolation and Identification of an α‐Glucosidase Inhibitory Peptide From Extruded Soybean Protein and Its Hypoglycemic Activity in T2DM Mice.” Food & Function 14, no. 9: 4288–4301. 10.1039/d3fo00580a. 37074029

[fsn371790-bib-0183] Li, X. , S. Wang , Q. Li , et al. 2025. “A Rapid and Reversible Molecular ‘Switch’ Regulating Protein Expression in *Chlamydomonas reinhardtii* .” Plant, Cell & Environment 48: 3913–3924. 10.1111/pce.15360. 39838873

[fsn371790-bib-0083] Li, Y. , G. Aiello , E. M. A. Fassi , et al. 2021. “Investigation of *Chlorella pyrenoidosa* Protein as a Source of Novel Angiotensin I‐Converting Enzyme (ACE) and Dipeptidyl Peptidase‐IV (DPP‐IV) Inhibitory Peptides.” Nutrients 13, no. 5: 1624. 10.3390/nu13051624. 34066103 PMC8151766

[fsn371790-bib-0084] Li, Y. , Y. Fan , J. Liu , et al. 2022. “Identification, Characterization and in Vitro Activity of Hypoglycemic Peptides in Whey Hydrolysates From Rubing Cheese By‐Product.” Food Research International 164: 112382. 10.1016/j.foodres.2022.112382. 36737967

[fsn371790-bib-0086] Li, Z. , Y. He , H. He , et al. 2023. “Purification Identification and Function Analysis of ACE Inhibitory Peptide From Ulva Prolifera Protein.” Food Chemistry 401: 134127. 10.1016/j.foodchem.2022.134127. 36096005

[fsn371790-bib-0087] Long, Z. , D. Pei , X. Zhu , et al. 2025. “Characterizing Recombinant Protein and Its Fragmentation by Top‐Down Mass Spectrometry.” Journal of Pharmaceutical and Biomedical Analysis 267: 117157. 10.1016/j.jpba.2025.117157. 41014883

[fsn371790-bib-0088] Ma, X. , M. Liu , R. Li , et al. 2022. “Research Progress of Animal‐Derived Hypoglycemic Peptides.” Science and Technology of Food Industry 43, no. 22: 438–444. 10.13386/j.issn1002-0306.2021110003.

[fsn371790-bib-0089] Mao, Y. , M. Liu , K. Su , et al. 2025. “Extraction and Hydrolysis of *Chlamydomonas reinhardtii* Protein and in Vitro MAO‐A Inhibitory Activity of Hydrolysates.” Journal of Applied Phycology 37, no. 4: 2407–2418. 10.1007/s10811-025-03517-w.

[fsn371790-bib-0090] Martínez, R. , A. García Beltrán , G. Kapravelou , et al. 2024. “Nutritional and Functional Assessment of Haloarchaea and Microalgae From the Andalusian Shoreline: Promising Functional Foods With a High Nutritional Value.” Journal of Functional Foods 116: 106194. 10.1016/j.jff.2024.106194.

[fsn371790-bib-0091] Masi, A. , F. Leonelli , V. Scognamiglio , G. Gasperuzzo , A. Antonacci , and M. A. Terzidis . 2023. “ *Chlamydomonas reinhardtii* : A Factory of Nutraceutical and Food Supplements for Human Health.” Molecules 28, no. 3: 1185. 10.3390/molecules28031185. 36770853 PMC9921279

[fsn371790-bib-0092] Mayfield, S. P. , A. L. Manuell , S. Chen , et al. 2007. “ *Chlamydomonas reinhardtii* Chloroplasts as Protein Factories.” Current Opinion in Biotechnology 18, no. 2: 126–133. 10.1016/j.copbio.2007.02.001. 17317144

[fsn371790-bib-0093] Melgosa, R. , M. Marques , A. Paiva , et al. 2021. “Subcritical Water Extraction and Hydrolysis of Cod ( *Gadus morhua* ) Frames to Produce Bioactive Protein Extracts.” Food 10, no. 6: 1222. 10.3390/foods10061222/s1. PMC8228765 34071297

[fsn371790-bib-0094] Mijiti, Y. , S. Subuer , N. Ullah , et al. 2025. “Research on Cumin Peptides Using PBS Extraction and Their Multifunctional Bioactivities.” arXiv, arXiv2502. https://arxiv.org/abs/2502.08264.

[fsn371790-bib-0095] Mojica, L. , E. Gonzalez de Mejia , M. Á. Granados‐Silvestre , and M. Menjivar . 2017. “Evaluation of the Hypoglycemic Potential of a Black Bean Hydrolyzed Protein Isolate and Its Pure Peptides Using in Silico, in Vitro and in Vivo Approaches.” Journal of Functional Foods 31: 274–286. 10.1016/j.jff.2017.02.006.

[fsn371790-bib-0096] Mora, L. , and F. Toldrá . 2022. “Advanced Enzymatic Hydrolysis of Food Proteins for the Production of Bioactive Peptides.” Current Opinion in Food Science 49: 100973. 10.1016/j.cofs.2022.100973.

[fsn371790-bib-0097] Munawaroh, H. S. H. , G. G. Gumilar , C. R. Alifia , et al. 2020. “Photostabilization of Phycocyanin From *Spirulina* Platensis Modified by Formaldehyde.” Process Biochemistry 94: 297–304. 10.1016/j.procbio.2020.04.021.

[fsn371790-bib-0098] Musaimi, O. A. , and D. M. M. Jaradat . 2024. “Advances in Therapeutic Peptides Separation and Purification.” Separations 11, no. 8: 233. 10.3390/separations11080233.

[fsn371790-bib-0099] Naveed, M. , F. Nadeem , T. Mehmood , M. Bilal , Z. Anwar , and F. Amjad . 2021. “Protease—A Versatile and Ecofriendly Biocatalyst With Multi‐Industrial Applications: An Updated Review.” Catalysis Letters 151, no. 2: 307–323. 10.1007/s10562-020-03316-7.

[fsn371790-bib-0100] Neagu, A. , M. Jayathirtha , E. Baxter , et al. 2022. “Applications of Tandem Mass Spectrometry (MS/MS) in Protein Analysis for Biomedical Research.” Molecules 27, no. 8: 2411. 10.3390/molecules27082411. 35458608 PMC9031286

[fsn371790-bib-0101] Newsholme, P. , V. Cruzat , F. Arfuso , et al. 2014. “Nutrient Regulation of Insulin Secretion and Action.” Journal of Endocrinology 221, no. 3: R105–R120. 10.1530/JOE-13-0616. 24667247

[fsn371790-bib-0102] Ochoa‐Méndez, C. E. , I. Lara‐Hernández , L. M. González , et al. 2016. “Bioactivity of an Antihypertensive Peptide Expressed in *Chlamydomonas reinhardtii* .” Journal of Biotechnology 240: 76–84. 10.1016/j.jbiotec.2016.11.001. 27816654

[fsn371790-bib-0103] Olasehinde, O. R. , M. A. Acho , O. B. Afolabi , and R. O. Arise . 2023. “Peptide Hydrolysate of *Telfairia occidentalis* Hook f. Seed Protein Promotes Effective Glucose Homeostasis by Improving β‐Cell Dysfunction and Abating Carbohydrate Metabolic Disturbance in Diabetic Rats.” Journal of Food Biochemistry 2023, no. 1: 6652466. 10.1155/2023/6652466.

[fsn371790-bib-0104] Ortizo, R. G. G. , V. Sharma , M. L. Tsai , et al. 2024. “A Novel Deep Eutectic Solvent‐Based Green Extraction and Purification of DPP‐IV Inhibitory Peptides From tilapia ( *Oreochromis niloticus* ) Viscera Hydrolysate.” Food Bioscience 61: 104658. 10.1016/j.fbio.2024.104658.

[fsn371790-bib-0105] Ovchinnikova, T. V. 2019. “Structure, Function, and Therapeutic Potential of Marine Bioactive Peptides.” Marine Drugs 17, no. 9: 505. 10.3390/md17090505. 31466341 PMC6780686

[fsn371790-bib-0106] Penasa, R. , G. Licini , and C. Zonta . 2025. “Advances in Chiral Analysis: From Classical Methods to Emerging Technologies.” Chemical Society Reviews 54, no. 23: 10940–10955. 10.1039/d4cs01202j. 41114632

[fsn371790-bib-0107] Perçin, P. S. , and S. Karakaya . 2020. “Evaluation of Protein Profiles, Bioactivity, Allergenicity and Toxicity of Peptides Generated After in Silico Digestion of Common Wheat and Einkorn Wheat.” Turkish Journal of Agriculture—Food Science and Technology 8, no. 4: 901–911. 10.24925/turjaf.v8i4.901-911.3072.

[fsn371790-bib-0108] Phadnis, G. , and G. Prakash . 2024. “Chloroplast Expression of Myoglobin and Functional Characterization of *C. reinhardtii* Biomass for Alternative Meat Applications.” Algal Research 84: 103807. 10.1016/j.algal.2024.103807.

[fsn371790-bib-0109] Pipaliya, R. , B. Basaiawmoit , A. A. Sakure , et al. 2024. “Production and Characterization of Anti‐Hypertensive and Anti‐Diabetic Peptides From Fermented Sheep Milk With Anti‐Inflammatory Activity: In Vitro and Molecular Docking Studies.” Journal of the Science of Food and Agriculture 105, no. 8: 4096–4120. 10.1002/jsfa.13617. 38855927

[fsn371790-bib-0110] Qu, W. , X. Deng , T. Guo , C. Zhou , and H. Ma . 2025. “Enhancing the Encapsulation Efficiency, Bioavailability, and Antioxidant Efficacy of Quercetin and Maize Peptide by a Novel Multi‐Frequency Pulsed Ultrasonic‐Assisted Nanoliposomal Encapsulation System.” Food Research International 218: 116874. 10.1016/j.foodres.2025.116874. 40790677

[fsn371790-bib-0111] Radhakrishnan, D. P. , A. Kanakaraja , N. Krishnan , M. Sakthivelu , S. C. B. Gopinath , and R. Pachaiappan . 2024. “HPLC Purification of Antioxidant and Antibacterial Peptides From a Lichen ‘Parmotrema Perlatum (Huds.) M. Choisy’: Identification by LC‐MS/MS Peptide Mass Fingerprinting.” Biotechnology and Applied Biochemistry 71, no. 3: 627–640. 10.1002/bab.2563. 38311972

[fsn371790-bib-0112] Ramos‐Romero, S. , J. R. Torrella , T. Pagès , G. Viscor , and J. L. Torres . 2021. “Edible Microalgae and Their Bioactive Compounds in the Prevention and Treatment of Metabolic Alterations.” Nutrients 13, no. 2: 563. 10.3390/nu13020563. 33572056 PMC7916042

[fsn371790-bib-0113] Rasala, B. A. , M. Muto , P. A. Lee , et al. 2010. “Production of Therapeutic Proteins in Algae, Analysis of Expression of Seven Human Proteins in the Chloroplast of *Chlamydomonas reinhardtii* .” Plant Biotechnology Journal 8, no. 6: 719–733. 10.1111/j.1467-7652.2010.00503.x. 20230484 PMC2918638

[fsn371790-bib-0114] Ratnaningsih, E. , R. Reynard , K. Khoiruddin , I. G. Wenten , and R. Boopathy . 2021. “Recent Advancements of UF‐Based Separation for Selective Enrichment of Proteins and Bioactive Peptides—A Review.” Applied Sciences 11, no. 3: 1078. 10.3390/app11031078.

[fsn371790-bib-0115] Rosales‐Mendoza, S. , L. M. T. Paz‐Maldonado , and R. E. Soria‐Guerra . 2011. “ *Chlamydomonas reinhardtii* As a Viable Platform for the Production of Recombinant Proteins: Current Status and Perspectives.” Plant Cell Reports 31, no. 3: 479–494. 10.1007/s00299-011-1186-8. 22080228

[fsn371790-bib-0116] Sanchez‐Avila, X. , R. M. De Oliveira , S. Huang , et al. 2025. “Trends in Mass Spectrometry‐Based Single‐Cell Proteomics.” Analytical Chemistry 97, no. 11: 5893–5907. 10.1021/acs.analchem.5c00661. 40091206 PMC12003028

[fsn371790-bib-0117] Sarmadi, B. , F. Aminuddin , M. Hamid , N. Saari , A. Abdul‐Hamid , and A. Ismail . 2012. “Hypoglycemic Effects of Cocoa (* Theobroma cacao L*.) Autolysates.” Food Chemistry 134, no. 2: 905–911. 10.1016/j.foodchem.2012.02.202. 23107706

[fsn371790-bib-0118] Savova, M. S. , L. V. Mihaylova , D. Tews , M. Wabitsch , and M. I. Georgiev . 2023. “Targeting PI3K/AKT Signaling Pathway in Obesity.” Biomedicine & Pharmacotherapy 159: 114244. 10.1016/j.biopha.2023.114244. 36638594

[fsn371790-bib-0119] Scaife, M. A. , G. T. D. T. Nguyen , J. Rico , D. Lambert , K. E. Helliwell , and A. G. Smith . 2015. “Establishing *Chlamydomonas reinhardtii* as an Industrial Biotechnology Host.” Plant Journal 82, no. 3: 532–546. 10.1111/TPJ.12781. PMC4515103 25641561

[fsn371790-bib-0120] Scheen, A. J. 2022. “Clinical Pharmacology of Antidiabetic Drugs: What Can Be Expected of Their Use?” La Presse Médicale 52, no. 1: 104158. 10.1016/j.lpm.2022.104158. 36565754

[fsn371790-bib-0121] Shahrestanaki, M. K. , F. P. Arasi , and M. Aghaei . 2019. “IPP‐1 Controls Akt/CREB Phosphorylation Extension in A2a Adenosine Receptor Signaling Cascade in MIN6 Pancreatic β‐Cell Line.” European Journal of Pharmacology 850: 88–96. 10.1016/J.EJPHAR.2019.02.017. 30772395

[fsn371790-bib-0122] Shamriz, S. , and H. Ofoghi . 2016. “Outlook in the Application of *Chlamydomonas reinhardtii* Chloroplast as a Platform for Recombinant Protein Production.” Biotechnology and Genetic Engineering Reviews 32, no. 1–2: 92–106. 10.1080/02648725.2017.1307673. 28359189

[fsn371790-bib-0123] Shekoohi, N. , P. Harnedy‐Rothwell , S. Sharkey , et al. 2024. “Purification and Characterization of Multifunctional Peptides With in Situ Insulinotropic and Antioxidative Activity From a Blue Whiting ( *Micromesistius poutassou* ) Protein Hydrolysate.” Journal of Functional Foods 116: 106173. 10.1016/J.JFF.2024.106173.

[fsn371790-bib-0124] Siahbalaei, R. , G. Kavoosi , and M. Noroozi . 2021. “Protein Nutritional Quality, Amino Acid Profile, Anti‐Amylase and Anti‐Glucosidase Properties of Microalgae: Inhibition and Mechanisms of Action Through in Vitro and in Silico Studies.” LWT 150: 112023. 10.1016/J.LWT.2021.112023.

[fsn371790-bib-0125] Sim, E. J. , Q. Tran , Y. R. Lee , et al. 2025. “Cell‐Penetrating Peptide‐Based Triple Nanocomplex Enables Efficient Nuclear Gene Delivery in *Chlamydomonas reinhardtii* .” Biotechnology and Bioengineering 122, no. 8: 2218–2227. 10.1002/bit.29019. 40342143 PMC12235220

[fsn371790-bib-0126] Song, G. , Y. Huang , M. Xiong , et al. 2021. “Aloperine Relieves Type 2 Diabetes Mellitus via Enhancing GLUT4 Expression and Translocation.” Frontiers in Pharmacology 11: 561956. 10.3389/FPHAR.2020.561956. 33568989 PMC7868325

[fsn371790-bib-0127] Soto‐Sierra, L. , P. Stoykova , and Z. L. Nikolov . 2018. “Extraction and Fractionation of Microalgae‐Based Protein Products.” Algal Research 36: 175–192. 10.1016/J.ALGAL.2018.10.023.

[fsn371790-bib-0128] Sridhar, K. , B. S. Inbaraj , and B. H. Chen . 2021. “Recent Developments on Production, Purification and Biological Activity of Marine Peptides.” Food Research International 147: 110468. 10.1016/J.FOODRES.2021.110468. 34399466

[fsn371790-bib-0129] Stožer, A. , E. P. Leitgeb , V. Pohorec , et al. 2021. “The Role of cAMP in beta Cell Stimulus–Secretion and Intercellular Coupling.” Cells 10, no. 7: 1658. 10.3390/cells10071658. 34359828 PMC8304079

[fsn371790-bib-0130] Su, K. , L. Wu , Y. Lin , et al. 2025. “Bioactive Peptides From *Chlamydomonas reinhardtii* Protein Hydrolysate: Identification, Antimicrobial Activity, and Mechanism of Action.” Food Chemistry: X 31: 103140. 10.1016/j.fochx.2025.103140. 41140584 PMC12552568

[fsn371790-bib-0131] Subirats, X. , and M. Rosés . 2025. “Characterization of HPLC Columns: A Comparison of Tanaka and Abraham Methods.” Journal of Chromatography A 1762: 466376. 10.1016/j.chroma.2025.466376. 40974701

[fsn371790-bib-0132] Sujitha, P. , and C. Shanthi . 2023. “Importance of Enzyme Specificity and Stability for the Application of Proteases in Greener Industrial Processing—A Review.” Journal of Cleaner Production 425: 138915. 10.1016/J.JCLEPRO.2023.138915.

[fsn371790-bib-0133] Suo, Q. , Y. Yue , J. Wang , N. Wu , L. Geng , and Q. Zhang . 2026. “Discovery and Molecular Mechanism of a Novel Antihypertensive Peptide From *Chlamydomonas reinhardtii* Based on Molecular Docking, Molecular Dynamics Simulation, in Vitro, and in Vivo Analysis.” Food Research International 22: 9118477. 10.1016/j.foodres.2026.118477. 41763799

[fsn371790-bib-0134] Tagliamonte, S. , V. Oliviero , and P. Vitaglione . 2024. “Food Bioactive Peptides: Functionality Beyond Bitterness.” Nutrition Reviews 83, no. 2: 369–381. 10.1093/nutrit/nuae008. 38350613

[fsn371790-bib-0135] Taneera, J. , and M. M. Saber‐Ayad . 2023. “Preservation of β‐Cells as a Therapeutic Strategy for Diabetes.” Hormone and Metabolic Research 56, no. 4: 261–271. 10.1055/A-2239-2668/ID/R2023-08-0347-0014/BIB. 38387480

[fsn371790-bib-0136] Tapadia, M. , R. Carlessi , S. Johnson , R. Utikar , and P. Newsholme . 2019. “Lupin Seed Hydrolysate Promotes G‐Protein‐Coupled Receptor, Intracellular Ca2+ and Enhanced Glycolytic Metabolism‐Mediated Insulin Secretion From BRIN‐BD11 Pancreatic beta Cells.” Molecular and Cellular Endocrinology 480: 83–96. 10.1016/J.MCE.2018.10.015. 30347229

[fsn371790-bib-0138] Tavano, O. L. 2013. “Protein Hydrolysis Using Proteases: An Important Tool for Food Biotechnology.” Journal of Molecular Catalysis B: Enzymatic 90: 1–11. 10.1016/J.MOLCATB.2013.01.011.

[fsn371790-bib-0137] Tavano, O. L. , A. Berenguer‐Murcia , F. Secundo , and R. Fernandez‐Lafuente . 2018. “Biotechnological Applications of Proteases in Food Technology.” Comprehensive Reviews in Food Science and Food Safety 17, no. 2: 412–436. 10.1111/1541-4337.12326. 33350076

[fsn371790-bib-0139] Tazon, A. W. , N. Mérindol , E. Fantino , et al. 2026. “Engineering Bidirectional Chloroplast Promoters for Tunable Co‐Expression of Multiple Genes in Microalgae ( *Chlamydomonas reinhardtii* ).” Communications Biology 9, no. 1: 202. 10.1038/s42003-025-09478-7. 41484447 PMC12891720

[fsn371790-bib-0140] Thongcumsuk, B. , W. Woraprayote , T. Janyaphisan , S. Cheunkar , and S. Oaew . 2023. “Microencapsulation and Peptide Identification of Purified Bioactive Fraction From *spirulina* Protein Hydrolysates With Dipeptidyl Peptidase IV (DPP‐IV) Inhibitory Activity.” Food Bioscience 56: 103438. 10.1016/J.FBIO.2023.103438.

[fsn371790-bib-0141] Tran, D. Q. , E. H. Ramos , and D. D. Belsham . 2016. “Induction of Gnrh mRNA Expression by the ω‐3 Polyunsaturated Fatty Acid Docosahexaenoic Acid and the Saturated Fatty Acid Palmitate in a GnRH‐Synthesizing Neuronal Cell Model, mHypoA‐GnRH/GFP.” Molecular and Cellular Endocrinology 426: 125–135. 10.1016/j.mce.2016.02.019. 26923440

[fsn371790-bib-0142] Uchida, M. , Y. Ohshiba , and O. Mogami . 2011. “Novel Dipeptidyl Peptidase‐4–Inhibiting Peptide Derived From β‐Lactoglobulin.” Journal of Pharmacological Sciences 117, no. 1: 63–66. 10.1254/JPHS.11089SC. 21836374

[fsn371790-bib-0143] Urošević, T. , and K. Trivunac . 2020. “Achievements in Low‐Pressure Membrane Processes Microfiltration (MF) and Ultrafiltration (UF) for Wastewater and Water Treatment.” In Elsevier eBooks, 67–107. 10.1016/b978-0-12-817378-7.00003-3.

[fsn371790-bib-0144] Valencia, P. , K. Espinoza , A. Ceballos , M. Pinto , and S. Almonacid . 2015. “Novel Modeling Methodology for the Characterization of Enzymatic Hydrolysis of Proteins.” Process Biochemistry 50, no. 4: 589–597. 10.1016/j.procbio.2014.12.028.

[fsn371790-bib-0145] Valencia‐Mejía, E. , K. A. Batista , J. J. A. Fernández , and K. F. Fernandes . 2019. “Antihyperglycemic and Hypoglycemic Activity of Naturally Occurring Peptides and Protein Hydrolysates From Easy‐To‐Cook and Hard‐To‐Cook Beans ( *Phaseolus vulgaris* L.).” Food Research International 121: 238–246. 10.1016/j.foodres.2019.03.043. 31108745

[fsn371790-bib-0146] Van Der Bruggen, B. 2018. “Microfiltration, Ultrafiltration, Nanofiltration, Reverse Osmosis, and Forward Osmosis.” In Elsevier eBooks, 25–70. 10.1016/b978-0-12-813483-2.00002-2.

[fsn371790-bib-0147] Van Heck, J. I. P. , M. Ajie , L. A. B. Joosten , et al. 2024. “Circulating Inflammatory Proteins Are Elevated in Type 1 and Type 2 Diabetes and Associated to Complications.” Diabetes, Obesity and Metabolism 27, no. 2: 719–728. 10.1111/dom.16066. PMC11701194 39562286

[fsn371790-bib-0148] Vilcacundo, R. , C. Martínez‐Villaluenga , and B. Hernández‐Ledesma . 2017. “Release of Dipeptidyl Peptidase IV, α‐Amylase and α‐Glucosidase Inhibitory Peptides From Quinoa ( *Chenopodium quinoa* Willd.) During in Vitro Simulated Gastrointestinal Digestion.” Journal of Functional Foods 35: 531–539. 10.1016/J.JFF.2017.06.024.

[fsn371790-bib-0149] Virolainen, P. A. , and E. M. Chekunova . 2024. “Transgenesis in Microalga *Chlamydomonas reinhardtii* : Current Approaches.” Ecological Genetics 22, no. 1: 47–62. 10.17816/ecogen624418.

[fsn371790-bib-0150] Wan, P. , B. Cai , H. Chen , et al. 2023. “Antidiabetic Effects of Protein Hydrolysates From Trachinotus ovatus and Identification and Screening of Peptides With α‐Amylase and DPP‐IV Inhibitory Activities.” Current Research in Food Science 6: 100446. 10.1016/J.CRFS.2023.100446. 36816000 PMC9932700

[fsn371790-bib-0153] Wang, J. , T. Wu , L. Fang , et al. 2020. “Anti‐Diabetic Effect by Walnut ( *Juglans mandshurica* Maxim.)‐derived Peptide LPLLR Through Inhibiting α‐Glucosidase and α‐Amylase, and Alleviating Insulin Resistance of Hepatic HepG2 Cells.” Journal of Functional Foods 69: 103944. 10.1016/J.JFF.2020.103944.

[fsn371790-bib-0154] Wang, K. , D. W. Sun , H. Pu , and Q. Wei . 2017. “Principles and Applications of Spectroscopic Techniques for Evaluating Food Protein Conformational Changes: A Review.” Trends in Food Science & Technology 67: 207–219. 10.1016/J.TIFS.2017.06.015.

[fsn371790-bib-0155] Wang, S. , L. Ma , J. Ji , et al. 2023. “Protective Effect of Soy Isolate Protein Against Streptozotocin Induced Gestational Diabetes Mellitus via TLR4/MyD88/NF‐κB Signaling Pathway.” Biomedicine & Pharmacotherapy 168: 115688. 10.1016/J.BIOPHA.2023.115688. 37890205

[fsn371790-bib-0156] Wang, T. , N. Bo , G. Sha , et al. 2024. “Identification and Molecular Mechanism of Novel Hypoglycemic Peptide in Ripened Pu‐Erh Tea: Molecular Docking, Dynamic Simulation, and Cell Experiments.” Food Research International 194: 114930. 10.1016/j.foodres.2024.114930. 39232541

[fsn371790-bib-0157] Wang, T. Y. , C. H. Hsieh , C. C. Hung , C. L. Jao , M. C. Chen , and K. C. Hsu . 2015. “Fish Skin Gelatin Hydrolysates as Dipeptidyl Peptidase IV Inhibitors and Glucagon‐Like Peptide‐1 Stimulators Improve Glycaemic Control in Diabetic Rats: A Comparison Between Warm‐ and Cold‐Water Fish.” Journal of Functional Foods 19: 330–340. 10.1016/J.JFF.2015.09.037.

[fsn371790-bib-0158] Wang, X. , Y. Deng , P. Xie , et al. 2023. “Novel Bioactive Peptides From *ginkgo biloba* Seed Protein and Evaluation of Their α‐Glucosidase Inhibition Activity.” Food Chemistry 404: 134481. 10.1016/J.FOODCHEM.2022.134481. 36240562

[fsn371790-bib-0160] Wicik, Z. , A. Nowak , J. Jarosz‐Popek , et al. 2022. “Characterization of the SGLT2 Interaction Network and Its Regulation by SGLT2 Inhibitors: A Bioinformatic Analysis.” Frontiers in Pharmacology 13. 10.3389/FPHAR.2022.901340/FULL. PMC9421436 36046822

[fsn371790-bib-0161] Xie, Y. , J. Wang , S. Wang , et al. 2024. “Preparation, Characterization, and Mechanism of DPP‐IV Inhibitory Peptides Derived From Bactrian Camel Milk.” International Journal of Biological Macromolecules 277: 134232. 10.1016/j.ijbiomac.2024.134232. 39098667

[fsn371790-bib-0162] Xie, Z. , Z. Chen , S. Wang , et al. 2025. “ *Chlamydomonas reinhardtii* (Red) Rich in Protoporphyrin IX Exerts Anti‐Diabetic Effects in Liver Tissue.” Molecular Nutrition & Food Research 69, no. 19: e70138. 10.1002/mnfr.70138. 40522037

[fsn371790-bib-0163] Xue, S. , L. Yang , M. Xu , Y. Zhang , and H. Liu . 2024. “The Screening of α‐Glucosidase Inhibitory Peptides From β‐Conglycinin and Hypoglycemic Mechanism in HepG2 Cells and Zebrafish Larvae.” International Journal of Biological Macromolecules 278: 134678. 10.1016/J.IJBIOMAC.2024.134678. 39137852

[fsn371790-bib-0164] Yan, Y. , Y. Li , Z. Zhang , et al. 2021. “Advances of Peptides for Antibacterial Applications.” Colloids and Surfaces. B, Biointerfaces 202: 111682. 10.1016/j.colsurfb.2021.111682. 33714188

[fsn371790-bib-0165] Yang, D. , C. Li , L. Li , et al. 2022. “Discovery and Functional Mechanism of Novel Dipeptidyl Peptidase IV Inhibitory Peptides From Chinese Traditional Fermented Fish (Chouguiyu).” Current Research in Food Science 5: 1676–1684. 10.1016/J.CRFS.2022.09.025. 36204708 PMC9529664

[fsn371790-bib-0166] Yang, J. , J. Hong , A. Aihaiti , et al. 2024. “Preparation of Sea Buckthorn ( *Hippophae rhamnoides* L.) Seed Meal Peptide by Mixed Fermentation and Its Effect on Volatile Compounds and Hypoglycemia.” Frontiers in Nutrition 11: 1355116. 10.3389/fnut.2024.1355116. 38414486 PMC10896959

[fsn371790-bib-0167] Yi, J. , J. Zhang , X. Chen , et al. 2026. “Heterotrophic Fermentation of a Robust Human Defensin in *Chlamydomonas reinhardtii* Provides a Stable and Potent Antibacterial.” Microbial Cell Factories 25, no. 1: 35. 10.1186/s12934-025-02916-5. 41486159 PMC12870362

[fsn371790-bib-0168] Yin, L. , S. Fu , R. Wu , et al. 2020. “Chain Conformation of an Acidic Polysaccharide From Green Tea and Related Mechanism of α‐Amylase Inhibitory Activity.” International Journal of Biological Macromolecules 164: 1124–1132. 10.1016/J.IJBIOMAC.2020.07.125. 32682045

[fsn371790-bib-0169] Yu, Y. , P. Sun , Y. Liu , et al. 2024. “Characterization and Evaluation of the in Vitro and in Vivo Anti‐Diabetic Activities of Camel Milk Protein Hydrolysates Derived With Different Protease Digestions.” Journal of Functional Foods 117: 106227. 10.1016/J.JFF.2024.106227.

[fsn371790-bib-0170] Yuan, X. , X. Gu , and J. Tang . 2008. “Purification and Characterisation of a Hypoglycemic Peptide From *Momordica charantia* L. Var. Abbreviata Ser.” Food Chemistry 111, no. 2: 415–420. 10.1016/j.foodchem.2008.04.006. 26047444

[fsn371790-bib-0171] Yuan, X. , P. Li , Z. Xiao , et al. 2023. “Preparation and Identification of Hypoglycemic Bioactive Peptide From *Amygdalus communis* L. by Multienzyme Hydrolysis.” Process Biochemistry 136: 292–300. 10.1016/j.procbio.2023.12.008.

[fsn371790-bib-0172] Yudhani, R. D. , Y. Sari , D. A. Nugrahaningsih , et al. 2023. “In Vitro Insulin Resistance Model: A Recent Update.” Journal of Obesity 2023, no. 1: 1964732. 10.1155/2023/1964732. 36714242 PMC9876677

[fsn371790-bib-0173] Zalucha, E. 2021. “The Role of GLP‐1 in the Regulation of Metabolism and Immune Responses.” Doctoral Dissertation, University of Michigan.

[fsn371790-bib-0175] Zhang, W. , M. A. Abubaker , Z. Li , et al. 2025. “Bioactive Peptides With Antioxidant and ACE Inhibitory Properties in Goat Milk Protein Hydrolysates: Peptidomics and Molecular Docking Study.” International Journal of Biological Macromolecules 299: 140286. 10.1016/j.ijbiomac.2025.140286. 39863228

[fsn371790-bib-0176] Zhao, C. , C. Yang , B. Liu , et al. 2018. “Bioactive Compounds From Marine Macroalgae and Their Hypoglycemic Benefits.” Trends in Food Science & Technology 72: 1–12. 10.1016/J.TIFS.2017.12.001.

[fsn371790-bib-0177] Zhou, H. , B. Safdar , H. Li , L. Yang , Z. Ying , and X. Liu . 2023. “Identification of a Novel α‐Amylase Inhibitory Activity Peptide From Quinoa Protein Hydrolysate.” Food Chemistry 403: 134434. 10.1016/J.FOODCHEM.2022.134434. 36358076

[fsn371790-bib-0178] Zhou, K. , T. Yu , M. Wan , et al. 2025. “High‐Density Heterotrophic Cultivation of a Cell‐Wall‐Deficient *Chlamydomonas reinhardtii* Strain by Fed‐Batch Strategy.” Biotechnology Letters 47, no. 4: 72. 10.1007/s10529-025-03614-3. 40610806

[fsn371790-bib-0179] Zhou, L. , C. Xiao , J. Gao , et al. 2024. “Preparation and Identification of Novel DPP‐IV Inhibitory Peptides From *Musculus senhousei* : Peptidomic Analysis, Molecular Simulation, and Validation.” Food Bioscience 59: 103832. 10.1016/J.FBIO.2024.103832.

[fsn371790-bib-0180] Zhou, L. H. , Z. G. Fu , P. Tan , et al. 2023. “Effect of *Chlamydomonas reinhardtii* Powder on Blood Glucose and Immune Function: A Randomized Clinical Trial.” International Journal of Frontiers in Medicine 5, no. 9: 1–4. 10.25236/IJFM.2023.050901.

[fsn371790-bib-0181] Zhu, Y. , R. Zheng , Z. Fan , et al. 2025. “Determination of 12 Halogenated Organic Pollutants in Edible Fish by Ultra Performance Liquid Chromatography‐High Resolution Mass Spectrometry Combined With Ultrasound‐Assisted Extraction and Gel Permeation Chromatography Purification.” Chinese Journal of Chromatography 43, no. 1: 68–77. 10.3724/sp.j.1123.2023.12028. 39722623 PMC11686473

[fsn371790-bib-0182] Zhu, Z. , H. Cao , X. Li , J. Rong , X. Cao , and J. Tian . 2021. “A Carbon Fixation Enhanced *Chlamydomonas reinhardtii* Strain for Achieving the Double‐Win Between Growth and Biofuel Production Under Non‐Stressed Conditions.” Frontiers in Bioengineering and Biotechnology 8: 603513. 10.3389/fbioe.2020.603513. 33511104 PMC7835968

