# Supplementary figures and images for: Mechanisms and Production of Hypoglycaemic Peptides: Exploring the Potential of Chlamydomonas reinhardtii
Source: Food Sci Nutr. 2026 Apr 14;14(4):e71790. doi: 10.1002/fsn3.71790 (PMC13079433; doi:10.1002/fsn3.71790)

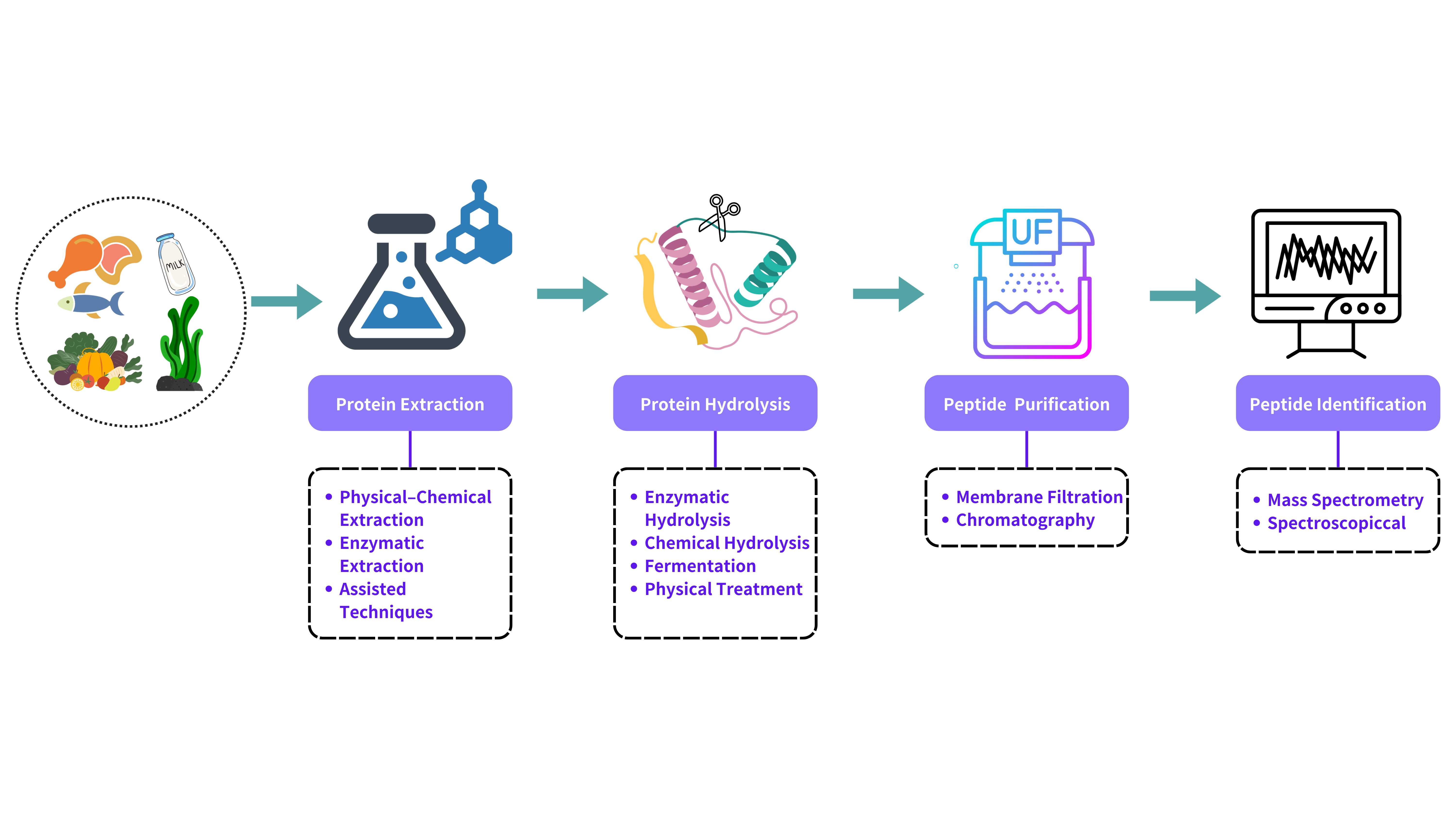

Supplement: Supplementary file 1 — Figure S1: Preparation workflow of bioactive peptides. [file FSN3-14-e71790-s002.png]
