# Supplementary material for: Mechanisms and Production of Hypoglycaemic Peptides: Exploring the Potential of Chlamydomonas reinhardtii
Source: Food Sci Nutr. 2026 Apr 14;14(4):e71790. doi: 10.1002/fsn3.71790 (PMC13079433; doi:10.1002/fsn3.71790)
Supplement: Supplementary file 2 — Table S1: Overview of methods for protein and peptide extraction, purification, identification, and bioactivity evaluation. [file FSN3-14-e71790-s001.docx]

Table S1. Overview of methods for protein and peptide extraction, purification, identification, and bioactivity evaluation

| **Category** | **Method** | **Principle** |
| --- | --- | --- |
| **Protein extraction** | |  |
|  | Physical–chemical extraction | Utilizes solvents, salts, pH adjustments, and other physicochemical approaches to modify protein solubility and enable extraction. Methods include solvent extraction, salt precipitation, and pH precipitation. |
|  | Enzymatic extraction | Employs specific enzymes, such as proteases or cell wall-degrading enzymes (e.g., pectinase, cellulase), to selectively hydrolyze cell walls or structural polysaccharides, disrupting cellular structures and releasing intracellular proteins. |
|  | Assisted techniques | Applies physical methods, including ultrasound, microwaves, high pressure, or supercritical fluids, to disrupt cells or enhance mass transfer, thereby promoting protein release and improving extraction efficiency. |
| **Protein hydrolysis** | |  |
|  | Enzymatic hydrolysis | Uses targeted proteases (e.g., Alcalase, trypsin, pepsin) under mild conditions to cleave peptide bonds selectively, generating bioactive peptides. |
|  | Chemical hydrolysis | Degrades peptide bonds through acid or alkali treatments, breaking proteins into smaller peptides or amino acids. |
|  | Fermentation | Leverages endogenous or microbial proteases produced during fermentation to progressively hydrolyze proteins into peptides. |
|  | Physical Treatment | Induces protein denaturation or fragmentation via thermal processing, high hydrostatic pressure, shear forces, pulsed electric fields, or irradiation, facilitating conversion into soluble low-molecular-weight products. |
| **Peptide purification** | |  |
|  | Membrane Filtration | Employs molecular weight cutoff membranes (e.g., microfiltration, ultrafiltration) to separate proteins and peptides by size, commonly used for crude fractionation and enrichment of low-molecular-weight peptides. |
|  | Chromatography | Separates and purifies peptides based on charge, hydrophobicity, or size using techniques such as ion exchange chromatography, reverse-phase high-performance liquid chromatography (RP-HPLC), or gel permeation chromatography. |
| **Peptide identification** | |  |
|  | Mass Spectrometry | Applies LC–MS/MS, MALDI-TOF, and related methods for precise determination of peptide molecular weight, amino acid sequencing, and quantitative analysis. |
|  | Spectroscopiccal | Uses UV-visible absorption, fluorescence spectroscopy, Fourier Transform Infrared Spectroscopy (FTIR), and Circular Dichroism (CD) to characterize peptide conformations, secondary structure, and intermolecular interactions. |
| **Bioactivity assay** | |  |
|  | Enzyme inhibition assays | Uses UV-visible absorption, fluorescence spectroscopy, Fourier Transform Infrared Spectroscopy (FTIR), and Circular Dichroism (CD) to characterize peptide conformations, secondary structure, and intermolecular interactions. |
|  | Cell-based assay | Evaluates peptide bioactivity at the cellular level using models such as HepG2, C2C12, or BRIN-BD11 cells, measuring glucose uptake, insulin secretion, signaling pathway modulation, and cytotoxicity. |
|  | Animal model assay | Examines the in vivo hypoglycemic effects of peptides, including regulation of pancreatic function, glucose tolerance, and overall metabolic homeostasis, using rodent models such as mice or rats. |
